# Elucidating AML ribosome biogenesis reshaping prognostic assessment and immune microenvironment integrated single-cell and bulk RNA analysis

**DOI:** 10.3389/fimmu.2025.1644671

**Published:** 2025-11-28

**Authors:** Xiaohe Chen, Aimei Feng, Fan Chen, Jue Zeng, Ming Chen

**Affiliations:** 1 Department of Blood Transfusion, The Third Affiliated Hospital of Wenzhou Medical University, Rui’an, China; 2 Department of Hematology, The Third Affiliated Hospital of Wenzhou Medical University, Rui’an, China

**Keywords:** acute myeloid leukemia, ribosome biogenesis, single-cell RNA sequencing, machine learning, diagnostic biomarkers, immune microenvironment

## Abstract

**Background:**

Acute Myeloid Leukemia (AML) is an aggressive hematologic malignancy with significant clinical challenges due to its heterogeneity and high relapse rate. Dysregulated ribosome biogenesis is a recognized driver of tumorigenesis and therapy resistance, but its comprehensive impact on AML prognosis and the immune microenvironment remains to be fully elucidated.

**Methods:**

We constructed an AML single-cell atlas and quantified ribosome biogenesis activity. A prognostic ribosome biogenesis signature (RBS) was developed through integrated machine learning and interpreted using the SHapley Additive exPlanations (SHAP) framework. We evaluated the RBS associations with prognosis, tumor immune microenvironment, immunotherapy response, and drug sensitivity. Key findings were validated by qPCR, Western blot, and molecular docking.

**Results:**

Single-cell analysis revealed significantly elevated ribosome biogenesis activity scores (RAS, a composite metric quantifying cellular ribosome synthesis capacity) in AML stem and progenitor cells. The RBS model, established using a Random Survival Forest algorithm, demonstrated robust prognostic power. SHAP interpretation identified EXOSC2 as the top predictor. Low-RBS patients displayed an immune-activated microenvironment and heightened immunotherapy response. Molecular docking indicated high-affinity binding of ouabain and digoxin to EXOSC2.

**Conclusion:**

Our study delineates the critical role of ribosome biogenesis in AML progression and establishes an interpretable RBS prognostic signature. This tool effectively assesses immunotherapy responsiveness and reveals novel targeting opportunities, providing valuable insights for clinical decision-making to improve AML outcomes.

## 1 Introduction

Acute Myeloid Leukemia (AML), an aggressive hematologic malignancy arising from hematopoietic stem/progenitor cells, manifests through differentiation arrest, aberrant proliferation, and dysregulated apoptosis in myeloid precursors (1, 2). This disease affects over 200,000 individuals globally each year, while maintaining an overall 5-year survival rate below 30%. Particularly adverse outcomes occur in elderly patients and those with high-risk genetic alterations such as FLT3-ITD and TP53 mutations (3–5). Current therapeutic advances—including targeted therapies (e.g., IDH inhibitors, BCL-2 antagonists) and immunotherapy—have not overcome limitations of standard intensive chemotherapy (anthracycline/cytarabine regimens) followed by allogeneic stem cell transplantation. Key challenges persist in treatment resistance/relapse driven by tumor heterogeneity, therapy-related toxicity, and immune evasion mediated by dysregulated microenvironmental interactions. Consequently, primary resistance affects 30%-40% of patients, and complete remission rates fall below 30% in high-risk subtypes (6–8). Our understanding of AML pathogenesis demands deeper insights into epigenetic regulation, metabolic reprogramming, and tumor-microenvironment crosstalk to enable next-generation therapies.

Within eukaryotic cells, ribosome biogenesis (RiboSis) constitutes a fundamental process initiated by RNA Pol I/III-mediated rRNA transcription in the nucleolus. This multistep pathway involves pre-rRNA splicing, chemical modifications (including pseudouridylation), stepwise assembly with ~79 ribosomal proteins (RPs), and nuclear export to generate functional cytoplasmic ribosomes (9–12). Over 200 assembly factors orchestrate this cascade, critically controlling cell proliferation and homeostasis (13). The emergent “Oncoribosome” concept underscores tumor-associated heterogeneity in ribosome composition and function (14, 15). Onco-ribosomes can originate from diverse alterations, including somatic mutations in ribosomal proteins (RPs) such as RPL10 and RPS15, which rewire the translatome to overexpress key oncogenes, or from disrupted rRNA modifications like the loss of conserved m¹acp³Ψ. In contrast to the uniform fidelity of normal ribosomes, this reprogramming enables selective translation of specific mRNA subsets, thereby activating tumorigenic pathways (16, 17). Dysregulated RiboSis—linked to malignancy and therapy resistance—fuels oncogenesis by compromising translation fidelity, inducing genomic instability, and activating pro-survival pathways, thereby establishing its therapeutic relevance (18–20). In AML specifically, ribosomes function dually as disease drivers and therapeutic targets: For instance, FBL-mediated rRNA 2’-O-methylation reprograms translation to favor amino acid transporter synthesis, elevating intracellular amino acid levels to drive leukemia stem cell (LSC) self-renewal (21). Contrastingly, KMT2D loss amplifies RiboSis via mTOR activation, simultaneously promoting leukemogenesis and sensitizing cells to RNA Pol I inhibitors like CX-5461 (22). Moreover, induced RiboSis defects (achieved through eIF6 overexpression) selectively eradicate leukemia cells, inhibit proliferation, and extend survival independently of p53 (23). Nevertheless, RiboSis impacts on the AML immune microenvironment, therapy resistance, and clinical outcomes remain incompletely defined. Notably, studies of ribosomopathies—inherited disorders of ribosome biogenesis with high AML risk—suggest that ribosomal defects can impair immune cell function, fostering a permissive microenvironment for leukemogenesis (24). This underscores the need to evaluate RiboSis in AML not only within leukemic blasts but also in the context of immune surveillance.

Technological innovations in single-cell RNA sequencing (scRNA-seq) have revolutionized AML research by resolving its inherent complexity (25). This approach provides high-resolution mapping of cellular heterogeneity, facilitating identification of key leukemia stem cell (LSC) subpopulations, their differentiation trajectories, and dynamic tumor microenvironment composition (26). Decoding these elements proves essential for discovering novel biomarkers, assessing patient status, predicting prognosis/therapy response, and elucidating AML pathogenesis, resistance mechanisms, and personalized therapeutic strategies (27, 28).

Our study extends previous findings that identified individual ribosome biogenesis factors like NCL as prognostic markers in AML through bulk analyses (29). However, we significantly advance this field by employing single-cell RNA sequencing, which allows us to precisely define ribosome biogenesis dysregulation specifically within hematopoietic stem and progenitor cells—the cellular compartments driving the disease. In contrast to single-gene biomarkers, we further integrated this knowledge to develop a novel, multi-gene ribosome biogenesis signature (RBS) using machine learning for robust prognostic stratification.

## 2 Methods

### 2.1 Data acquisition

AML single-cell RNA sequencing (scRNA-seq) data were obtained from the Zenodo database (https://doi.org/10.5281/zenodo.3345981) (30), comprising nine samples (5 AML bone marrow aspirates and 4 healthy donor [HD] controls). For independent single-cell validation, we utilized the ‘AML_GSE116256’ dataset from the Tumor Immune Single-cell Hub 2 (TISCH2) database (http://tisch.compbio.cn/home/) (31). This dataset profiles bone marrow samples from 16 adult patients with *de novo* AML. We included all 16 patients from this study in our analysis to ensure a comprehensive validation cohort that captures the cellular heterogeneity of AML. Bulk RNA-seq data and clinical information were downloaded from the GEO database. To ensure platform consistency and optimal genomic coverage for profiling ribosome biogenesis genes, we extracted data specifically from the GPL570 platform (Affymetrix U133 Plus 2.0 Array) for both the GSE37642 and GSE12417 series. Raw CEL files were processed using the Robust Multiarray Averaging (RMA) algorithm, and after selecting samples with available overall survival (OS) data, we obtained 136 patients from GSE37642 and 79 from GSE12417. These datasets were merged and batch-corrected using the ComBat function from the sva R package (v3.52.0) (**Supplementary Figure S1**), resulting in a combined cohort of 21,873 genes across 215 samples. For independent validation, we utilized the BeatAML program (https://biodev.github.io/BeatAML2/) (32) (439 patients after excluding non-AML malignancies), the TCGA_LAML dataset (https://portal.gdc.cancer.gov/) (132 patients with OS), and the OHSU AML cohort (Cancer Cell 2022, https://www.cbioportal.org/study/summary?id=aml_ohsu_2022) (513 subjects with survival and mutational data). These RNA-seq cohorts were analyzed using their provided TPM values without additional normalization. A RiboSis gene set of 331 genes was curated from the Molecular Signatures Database (MSigDB) Gene Ontology (GO) term and published characterization by Nerurkar et al. (**Supplementary Table 1**) (12, 33). Among these, 287 genes showed detectable expression in the merged bulk RNA-seq dataset.

### 2.2 Single-cell data processing and annotation

Single-cell RNA-seq data from both the primary and independent validation (GSE116256) datasets were processed using Seurat (v5.0.1) in R (v4.4.1) under a unified analytical workflow. Briefly, quality control was performed by filtering out genes detected in fewer than 3 cells. Cells were retained if they expressed between 200 and 6,000 genes, with dataset-specific filters further applied: the primary cohort excluded cells with >20% mitochondrial gene content or >100,000 total UMIs, and doublets were removed using DoubletFinder (v2.0.4), yielding 101,077 high-quality cells; the GSE116256 validation set excluded cells with mitochondrial content >10%, resulting in 30,659 high-quality cells for subsequent analysis.

The data from each dataset were then independently subjected to log-normalization, identification of highly variable genes, and dimensionality reduction via principal component analysis (PCA). Batch effects across samples were corrected using the RunHarmony function from the harmony R package (v1.2.1) with parameters group.by.vars = “orig.ident” and theta = 2. Cell clustering was performed using a graph-based algorithm, with resolutions set at 0.2 for the primary cohort and 0.5 for the validation set. Cell types in the primary cohort were annotated using canonical markers from an established AML scRNA atlas (34) and the CellMarker 2.0 database, while annotations for the GSE116256 dataset were cross-referenced with the TISCH2 database and marker genes.

### 2.3 Identification of cluster marker genes and pathway enrichment

Differentially expressed genes (DEGs) specific to each cell cluster were identified using FindAllMarkers in Seurat, with parameters: only.pos = TRUE (retain upregulated genes), min.pct = 0.25 (minimum expression fraction 25%), and logfc.threshold = 0.25 (absolute log-fold change >0.25). Gene Ontology Biological Process (GO-BP) and KEGG pathway enrichment analyses were performed on these DEGs using enrichCluster from clusterGVis (v0.1.2), with gene ID conversion facilitated by org.Hs.eg.db (v3.20.0). The top 5 enriched terms per pathway were extracted for visualization.

### 2.4 RiboSis activity scoring

Single-cell RiboSis activity scores (RAS) were computed using the “singscore,” “ssgsea,” and “viper” algorithms from the irGSEA package (v3.3.2). The scores from each algorithm were Z-score normalized and integrated via weighted principal component analysis (WPCA), with weights automatically derived from their variance contributions. The first principal component (PC1), explaining >70% of the total variance, was defined as the composite RAS. Based on the composite RAS (PC1) values, all cells (or samples) were classified into high- and low-RAS groups using the median value as the cutoff. The corresponding cell-level grouping results are provided in **Supplementary Materials**.

### 2.5 Copy number variation analysis

Clonal chromosomal copy number alterations in HSCs, GMPs, and MEPs were analyzed using InferCNV (v1.22.0) and CopyKAT (v1.1.0). For InferCNV, T/NK cells from the same AML samples were used as the reference to identify large-scale CNVs.

The CopyKAT analysis was applied to achieve higher-resolution detection of AML-specific alterations. Using T/NK cells as diploid references, CopyKAT was run on unmodified UMI counts with default parameters to estimate genomic copy number profiles. Cells were classified as aneuploid based on genome-wide CNA patterns. Cluster-level consensus CNA profiles were generated by averaging the discrete copy number calls (−1 for loss, +1 for gain, 0 for neutral) across all cells within the same cluster.

### 2.6 Functional and metabolic pathway analysis of high/low RAS groups

HALLMARK pathway enrichment analysis between high/low RAS groups used GSVA (v2.0.0). Pathway activity scores were calculated via single-sample gene set enrichment analysis (ssGSEA) with gene sets containing 10–500 genes (minSize=10, maxSize=500), employing Gaussian kernel density estimation (kcdf=“Gaussian”) and maximum difference standardization (maxDiff=TRUE). Metabolic activity was assessed using AUCell in scMetabolism (v0.2.1) to score KEGG metabolic pathways at single-cell resolution, retaining raw expression matrices (imputation=FALSE). Heatmaps visualized differential pathway and metabolic activity patterns between groups.

### 2.7 Cell communication analysis

CellChat (v2.1.2) analyzed differential interaction networks between high/low RAS groups. Cell-cell communication events were inferred through co-expression patterns of ligand-receptor pairs in secreted signaling pathways. The netVisual_aggregate function visualized global signal input/output strength, while netVisual_bubble identified significantly differential ligand-receptor interactions between groups (ligands: outgoing signals; receptors: incoming signals).

### 2.8 Identification of key ribosome biogenesis-related genes and consensus clustering

Forty RRGs significantly associated with overall survival (univariate Cox proportional hazards regression, p<0.05) were identified using the integrated bulk RNA-seq dataset. Consensus clustering of the RRG co-expression network used ConsensusClusterPlus (v1.68.0). The optimal cluster number (k=2) was determined by evaluating cumulative distribution function (CDF) curves from repeated subsampling and consensus matrix heatmap analysis. Principal component analysis (PCA) confirmed significant transcriptomic differences between subtypes. Prognostic disparity was assessed using Kaplan-Meier survival curves (survival v3.7-0) and log-rank tests.

### 2.9 Construction of RBS prognostic model via integrated machine learning

To develop a robust ribosome biogenesis signature (RBS), we integrated ten machine learning algorithms: Random Survival Forest (RSF), Least Absolute Shrinkage and Selection Operator (LASSO), Gradient Boosting Machine (GBM), Survival Support Vector Machine (Survival-SVM), Supervised Principal Components (SuperPC), Ridge Regression, Partial Least Squares Regression for Cox (plsRcox), CoxBoost, Stepwise Cox, and Elastic Net (Enet). The GSE37642 dataset served as the primary training cohort. For model development and selection, we employed a multi-step validation approach: both the GSE12417 dataset and a merged cohort (GSE37642+GSE12417) functioned as internal validation cohorts to evaluate 81 candidate models generated through algorithmic combinations. The optimal model was selected based on demonstrating the highest mean concordance index (C-index) across these internal validation cohorts. The BeatAML, TCGA_LAML and OHSU cohort was subsequently used as a fully independent external validation cohort to provide an unbiased assessment of the final model’s prognostic performance. Individualized risk scores were computed using the coefficients from the optimal model. For clinical stratification, patients were classified into high- and low-RBS subgroups using the optimal riskScore cutoff, determined by maximizing the survival difference between groups through the “surv_cutpoint” function in the R package survminer.

### 2.10 Prognostic validation and nomogram construction

Kaplan-Meier analysis (survminer) compared overall survival between high and low RBS subgroups using the log-rank test. Predictive accuracy at 1, 3, and 5 years was quantified using time-dependent ROC curves (timeROC), reported as AUC. An individualized nomogram integrated the RBS score with independent prognostic factors (e.g., age) to predict survival. This model’s performance was comprehensively evaluated: calibration curves assessed the agreement between predicted and observed survival probabilities; decision curve analysis (DCA) quantified the clinical net benefit across different threshold probabilities; and multi-timepoint ROC curves evaluated discriminative ability over time.

### 2.11 Explainable analysis based on the SHAP model

To enhance model interpretability, we employed the SHapley Additive exPlanations (SHAP) framework for attribution analysis of the optimal prognostic model. This model-agnostic approach, grounded in Shapley value theory, quantifies each feature’s contribution to individual predictions. For each sample prediction, the expected marginal contribution of a target feature across all possible feature subsets (i.e., the SHAP value) was calculated. We implemented approximate computation and visualization using KernelSHAP (shapviz v0.9.3) with default parameters. The analysis was performed independently for each validation cohort using the training set as reference background.

### 2.12 Pathway activity and functional enrichment analysis

Gene Set Variation Analysis (GSVA) and single-sample Gene Set Enrichment Analysis (ssGSEA) quantified pathway activity. Conventional Gene Set Enrichment Analysis (GSEA) interpreted biological functions using canonical gene sets from MsigDB. Core tools included GSVA, clusterProfiler, GSEABase, and gseaVis. Heatmaps displayed subtype-specific pathway activity; ridge plots characterized core pathway expression distributions. Permutation tests (1,000 iterations) validated enrichment significance.

### 2.13 Immune microenvironment profiling and treatment response prediction

We employed IOBR (v0.99.8) to integrate eight deconvolution algorithms (MCPcounter, EPIC, xCell, CIBERSORT, IPS, quanTIseq, ESTIMATE, TIMER) to quantitatively assess immune cell infiltration and microenvironment scores. Pearson/Spearman correlation assessed the association between RBS risk scores and immune checkpoint molecules, chemokines, and their receptors. Treatment response indicators were computed using TIDE (http://tide.dfci.harvard.edu/), focusing on four core immune evasion indicators: Immune Exclusion score, M2 Tumor-Associated Macrophages (TAM M2), Merck18 immune phenotype, and CD8+ T cell activity. Boxplots visualized differences between high- and low-risk groups.

### 2.14 Drug sensitivity analysis and small-molecule drug screening

Drug sensitivity (IC_50_) for AML samples was predicted using oncoPredict (v1.2) based on the GDSC database (https://www.cancerrxgene.org/). Higher IC_50_ values indicate lower sensitivity. To identify EXOSC2-targeting drugs, we screened DSigDB via Enrichr (https://maayanlab.cloud/Enrichr/) applying criteria: (1) adjusted P < 0.05; (2) odds ratio [OR] > 40; (3) explicit inclusion of EXOSC2. Ouabain showed the most significant association in the HL60 downregulation group (adjusted P = 0.009, OR = 106.87). Digoxin had borderline significance (adjusted P = 0.051) but OR (43.44) exceeded the threshold, and prior studies confirmed its specific mechanism in AML (35). Based on effect size and target specificity evidence, both were selected for docking.

### 2.15 Molecular docking model construction

We retrieved the SDF structures of ouabain and digoxin from PubChem. Their 3D structures were generated using ChemOffice 20.0. The EXOSC2 protein sequence (UniprotKB: Q13868) was obtained from UniProt. Molecular docking was performed using AutoDock Vina. Compound binding affinity was assessed based on calculated binding energies. Results were visualized using PyMOL 2.6.0 and Discovery Studio 2019.

### 2.16 Quantitative real-time PCR

This study was approved by the Ethics Committee of the Third Affiliated Hospital of Wenzhou Medical University (Approval No.: YJ2025005). Written informed consent was obtained from 8 AML patients and 8 healthy controls. Bone marrow mononuclear cells (BMMNCs) were isolated from fresh samples using Ficoll-Paque PLUS density gradient centrifugation. Total RNA was extracted with TRIzol reagent (Invitrogen) according to the manufacturer’s protocol, and RNA quality was assessed by spectrophotometry. cDNA was synthesized from 1 μg of total RNA using the PrimeScript RT Reagent Kit (Takara). Expression levels of four model genes were quantified by qRT-PCR on an ABI Q6 Real-Time PCR System (Applied Biosystems) with Acro Biotech SYBR Green kits. Relative gene expression was calculated using the 2^(–ΔΔCT) method, with GAPDH as the endogenous control. Primer sequences are provided in **Supplementary Table 2**.

### 2.17 Western blotting

Bone marrow samples were lysed in red blood cell lysis buffer (6 volumes). After centrifugation (800 × g, 10 min), the pellet was resuspended and lysed in RIPA buffer with protease/phosphatase inhibitors. Protein concentration was determined (Omni-Easy™ BCA Protein Assay Kit, Epizyme Biotech, Cat# ZJ102). Protein samples were mixed with loading buffer, denatured, and separated via SDS-PAGE. Proteins were transferred to PVDF membrane (wet transfer). The membrane was blocked (5% non-fat dry milk in TBST, 1 h, RT), incubated with primary antibodies (Proteintech) overnight (4°C), washed, incubated with HRP-conjugated secondary antibodies (2 h, RT), washed again, and developed using ECL substrate (Vazyme Biotech, Cat# E422-02). Signals were detected using a chemiluminescence imaging system. Band intensities were quantified using ImageJ.

### 2.18 Statistical analysis

Statistical analyses used R (v4.4.1). Spearman rank correlation assessed associations between continuous variables. Survival differences (high/low-risk groups) were analyzed using Kaplan-Meier curves (log-rank test). Group comparisons used Student’s t-test, Wilcoxon rank-sum test (two groups), ANOVA, or Kruskal-Wallis test (multiple groups), based on distribution. Univariate and multivariate Cox proportional hazards regression evaluated associations between risk score, clinical features, and overall survival (OS), reporting hazard ratios (HR) with 95% confidence intervals (CI). The Benjamini-Hochberg (BH) method controlled the false discovery rate. Statistical significance was defined as two-tailed P < 0.05 (q-value after correction).

## 3 Results

### 3.1 scRNA-seq analysis of AML

**Figure 1:**
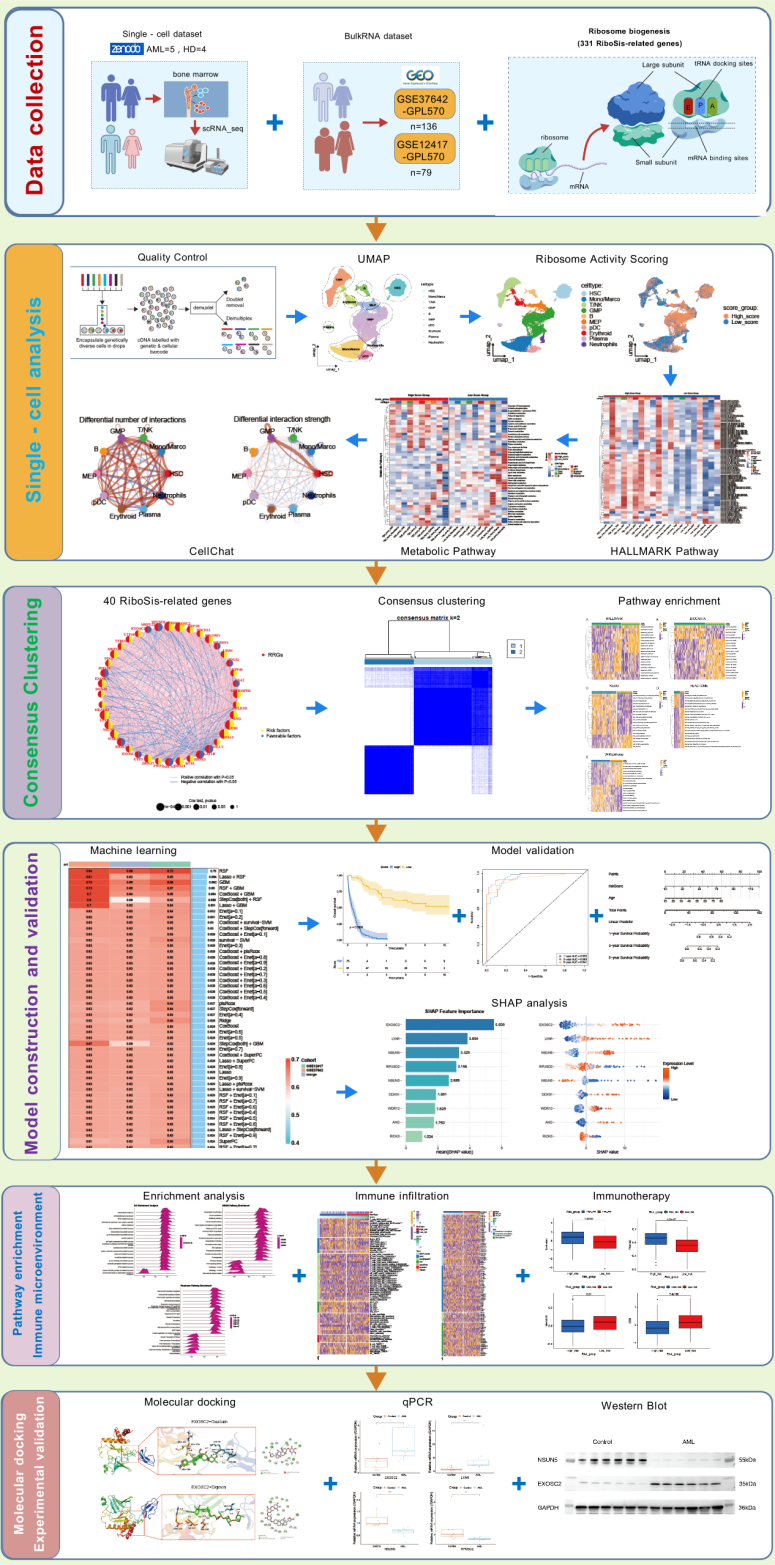
Flowchart of the research.

**Figure 2:**
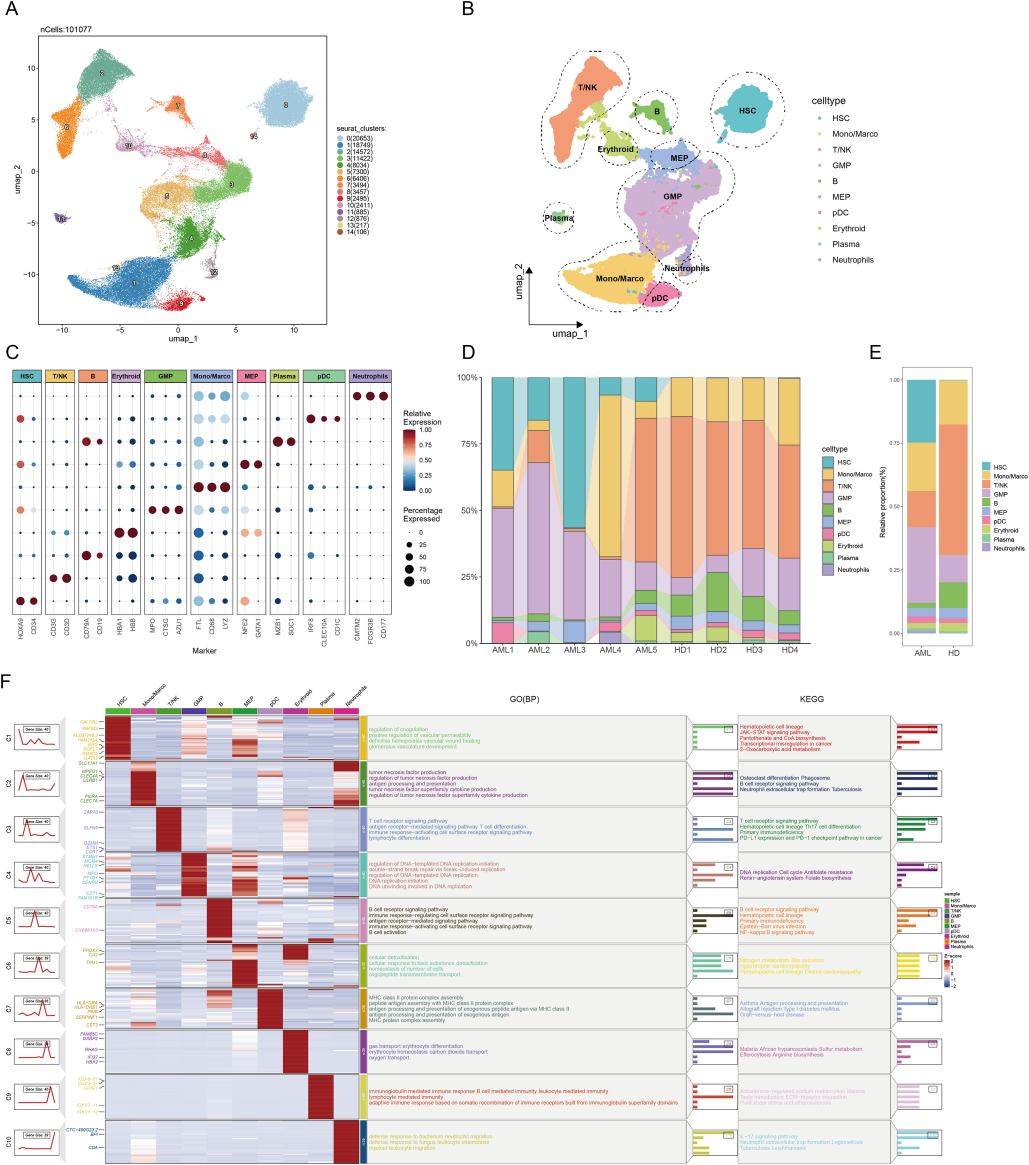
Integrated analysis of AML scRNA-seq data **(A)** Application of dimensionality reduction and clustering to cells from 5 AML and 4 HD resolved 15 cell clusters. **(B)** The UMAP visualization depicts 10 annotated cell types. **(C)** The bubble plot displays canonical marker genes for 10 cell subtypes. **(D)** Cell proportion plot across 9 samples. **(E)** Cell proportion plot: AML vs. HD. **(F)** The heatmap visualizes top DEGs across cell subtypes with parallel enrichment analysis for GO-BP and KEGG.

**Figure 1** illustrates the study workflow, generated using Biogdp (https://biogdp.com/) (36). Our analysis encompassed two distinct cohorts: a primary discovery cohort of 101,077 high-quality cells from Zenodo and an independent validation cohort of 30,659 cells from the GSE116256 dataset. Batch effects across nine samples in the discovery cohort were corrected using Harmony. Following UMAP dimensionality reduction and Leiden clustering, 15 distinct cell clusters were identified in the primary dataset (**Figure 2A**). These clusters were annotated into 10 cell types using canonical markers, including hematopoietic stem cells (HSC; HOXA9, CD34), granulocyte-macrophage progenitors (GMP; MPO, CTSG, AZU1), malignant cells, and other major immune/hematopoietic lineages (**Figure 2B**). In the validation set, cell types were directly visualized and annotated based on canonical markers, resulting in 13 annotated cell types as shown in the UMAP plot (**Supplementary Figure S2A**). A bubble plot visualizes marker gene expression across AML cell types in the primary cohort (**Figure 2C**), while a heatmap displays the top 3 differentially expressed genes among the 13 cell types in the validation set (**Supplementary Figure S2B**). Cell type distribution differed significantly between AML and normal samples (**Figures 2D, E**). GO and KEGG enrichment analysis of differentially expressed genes elucidated core biological functions of subpopulations (**Figure 2F**, **Supplementary Table 3**).

### 3.2 Characterization of RRGs in scRNA-seq data

**Figure 3:**
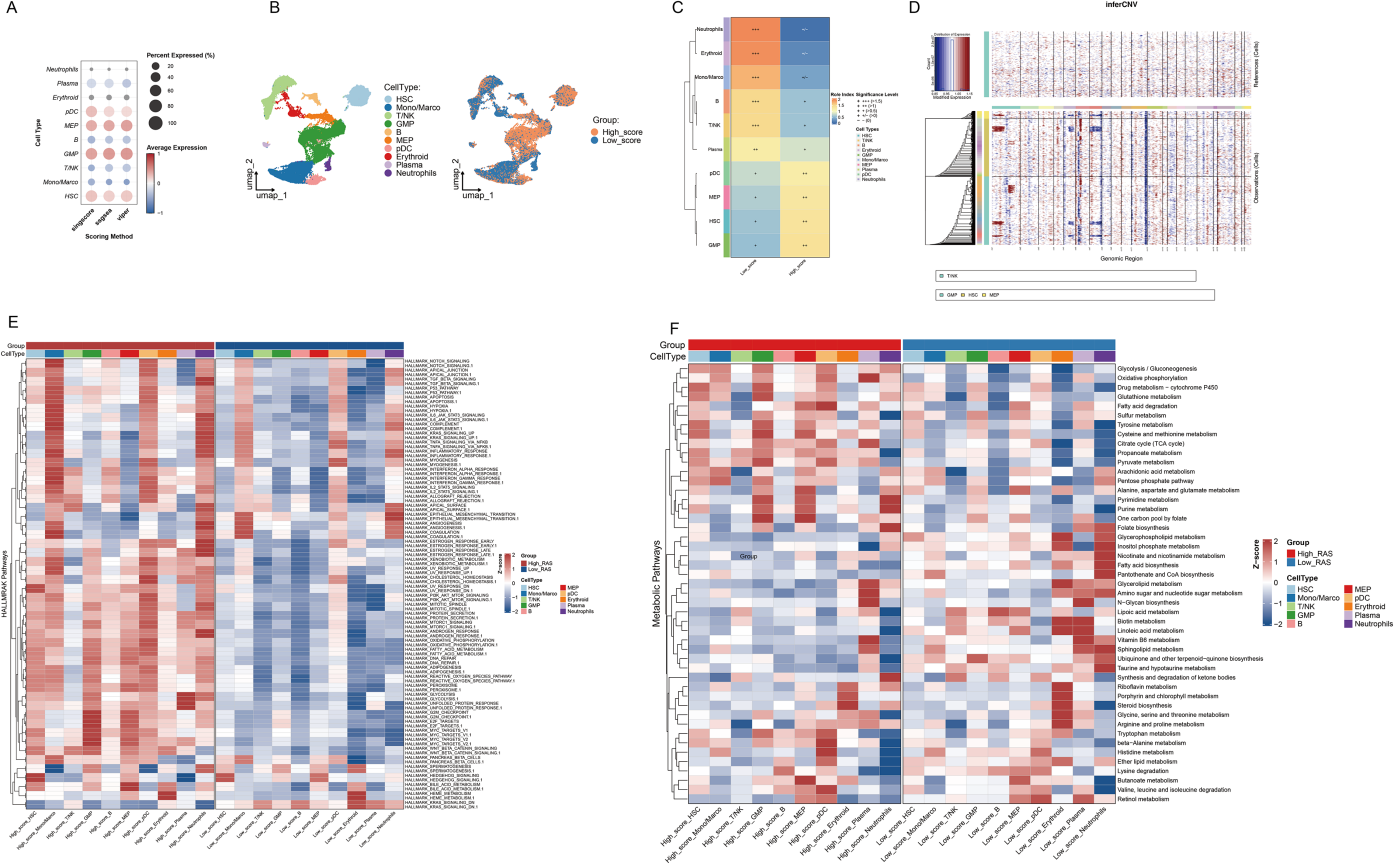
Profiling RiboSis-related genes in AML scRNA-seq. **(A)** Bubble plot comparing RiboSis activity scores across 10 cell subtypes using three algorithms. **(B)** The UMAP plot displays cell distribution in high and low RiboSis activity groups. **(C)** Tissue preference assessment of high/low RiboSis activity groups using the Ro/e index. **(D)** Copy number variation (CNV) inference in progenitor cells (HSCs/GMPs/MEPs) using T/NK cells as reference: Columns denote chromatin regions, rows represent cells. **(E)** The heatmap shows HALLMARK pathway scores (z-scores) across cell subpopulations in different RiboSis activity groups (red: high scores, blue: low scores). **(F)** Metabolic pathway scores across cell subpopulations by RiboSis activity group.

We first quantified the ribosome biogenesis activity at single-cell resolution using a Ribosome biogenesis Activity Score (RAS), a composite metric derived from the expression of 331 RRGs (see Methods). For subsequent comparative analyses, cells were stratified into high- and low-RAS groups based on the median RAS value, a threshold that ensures balanced group sizes for robust downstream comparisons. Analysis of 83,665 AML cells revealed significantly elevated RAS in HSCs, GMPs, and MEPs (**Figure 3A**). High- and low-RAS subpopulations showed distinct spatial segregation by UMAP (**Figure 3B**), validated by RO/e ratio (**Figure 3C**). InferCNV revealed increased genomic instability in HSCs/GMPs/MEPs compared to T/NK cells (**Figure 3D**), which was further supported by copyKAT analysis identifying recurrent deletions in high-risk chromosomal regions including del(5q) and -7/del(7q), but not chromosome 17 (**Supplementary Figures S3A–C**). These findings align with malignant progenitor expansion in AML and reinforce the association between RAS signaling and leukemic stemness. GSVA using HALLMARK gene sets demonstrated widespread pathway activation (50 pathways, e.g., G2M_CHECKPOINT, DNA_REPAIR, MYC_TARGETS) in high-RAS cells (**Figure 3E**). Conversely, low-RAS monocytes/macrophages/neutrophils showed limited pathway activation (e.g., inflammation), with downregulation in other subtypes.Metabolic scoring identified significant activation of 15 core pathways (including glycolysis, oxidative phosphorylation, purine metabolism) in high-RAS (HSCs/GMPs/MEPs) subpopulations (**Figure 3F**). This coordinated activation supports proliferation via ATP synthesis (Warburg effect) and provides nucleotide precursors/DNA replication support. Glutathione and cytochrome P450 pathway activation further suggests chemoresistance mechanisms via ROS scavenging/drug metabolism. In the validation cohort, elevated activity scores were predominantly observed in progenitor, precursor, and malignant cell populations (**Supplementary Figure S2C**). Cells were clearly segregated into high- and low-RAS subgroups by UMAP (**Supplementary Figure S2D**), and high-RAS malignant and progenitor cells exhibited consistent enrichment of proliferation-related pathways (e.g., E2F_TARGETS, G2M_CHECKPOINT) and key oncogenic programs (e.g., MYC_TARGETS_V1, MYC_TARGETS_V2) (**Supplementary Figure S2E**). Metabolic analysis further confirmed the skewed profile of high-RAS subsets toward energy production and biomass synthesis (e.g., glycolysis, oxidative phosphorylation), contrasting with the structural maintenance and specialized functional profiles seen in low-RAS cells (**Supplementary Figure S2F**). Collectively, high RAS correlates with key AML malignant features—increased genomic instability, accelerated cell cycle, and chemoresistance—implying a potential driving role in AML progression.

### 3.3 Differential cell-cell communication patterns between high and low RAS sample groups

**Figure 4:**
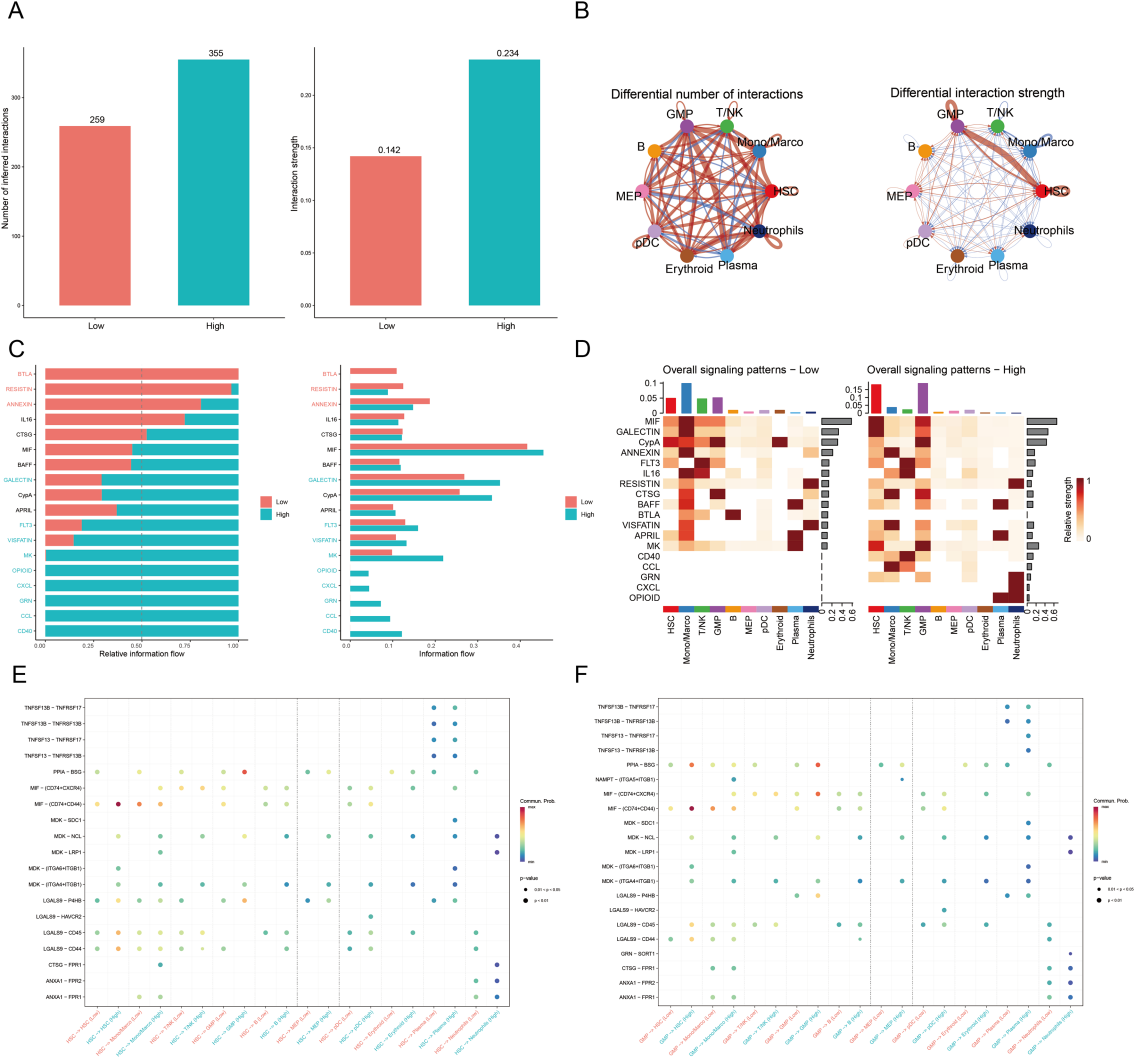
Comparative analysis of cell-cell interactions between high and low RiboSis activity groups. **(A)** The bar chart shows the total number (left) and weight (right) of ligand-receptor interactions between high- and low-RAS groups. **(B)** Differential communication network showing overall quantity and strength differences. Red lines: stronger in high-RAS group; blue lines: stronger in low-RAS group. **(C)** Comparison of pathway signaling activity indicating group-specific pathway enhancements in high- and low-RAS groups. **(D)** Heatmap depicting overall pathway signaling activity across high- and low-RAS groups, with pathways in rows and cell subsets in columns. Deepening color indicates increasing signaling strength. **(E)** Dot plot displays signaling activity changes in HSC relative to other cell types. **(F)** Dot plot displays signaling activity changes in GMP relative to other cell types.

CellChat analysis comparing high- and low-RAS sample groups revealed distinct cellular interaction networks, identifying 779 and 532 significant ligand-receptor interactions in the low- and high-activity groups, respectively (**Figure 4A**, **Supplementary Table 4**). This finding was further supported by analysis using cell-based stratification, which identified 259 and 355 interactions in the respective groups and similarly highlighted hematopoietic stem cells (HSCs) and granulocyte-macrophage progenitors (GMPs) as key mediators (**Supplementary Figures S4A, B**). Differential network analysis from the sample-level approach further confirmed HSCs and GMPs as the primary cellular populations driving inter-group heterogeneity, demonstrating coordinated alterations in both interaction number and strength (**Figure 4B**). Core pathway analysis revealed a distinct functional partition between groups. Homeostatic signaling pathways, including ANNEXIN and BTLA, were predominantly observed in the low-RAS group, whereas pro-malignant pathways such as Macrophage Migration Inhibitory Factor (MIF), CLEC, COLLAGEN, and CD99 were significantly enriched in the high-RAS group (**Figure 4C**). This pattern of pathway enrichment was corroborated by the cell-based stratification analysis, which similarly demonstrated a shift from homeostatic to pro-malignant signaling (**Supplementary Figure S4C**).

Analysis of cell-type-specific signaling patterns demonstrated that Monocytes/Macrophages and T/NK cells served as the primary signaling nodes in the low-RAS group, while HSCs and GMPs functioned as central communication hubs in the high-RAS network (**Figure 4D**). This cellular signaling hierarchy was largely conserved in the cell-based stratification analysis, with Monocytes/Macrophages (but not T/NK cells) and HSCs/GMPs remaining as the primary signaling nodes in the low- and high-RAS groups, respectively (**Supplementary Figure S4D**). Differential ligand-receptor interaction analysis from both analytical approaches consistently identified potent immunosuppressive signaling via the MIF-(CD74+CD44) axis, with particularly strong activity in HSC-HSC and HSC-GMP communication within the high-activity group (**Figures 4E, F**, **Supplementary Figures S4E, F**). Given that MIF-CD74 interactions are known to promote an immunosuppressive M2 phenotype in myeloid cells and facilitate immune evasion (37, 38), these results collectively demonstrate that high-RAS HSCs and GMPs may contribute to acute myeloid leukemia pathology through activation of the MIF-(CD74+CD44) axis.

### 3.4 AML subtyping based on rrgs and functional/immune microenvironment characterization

**Figure 5:**
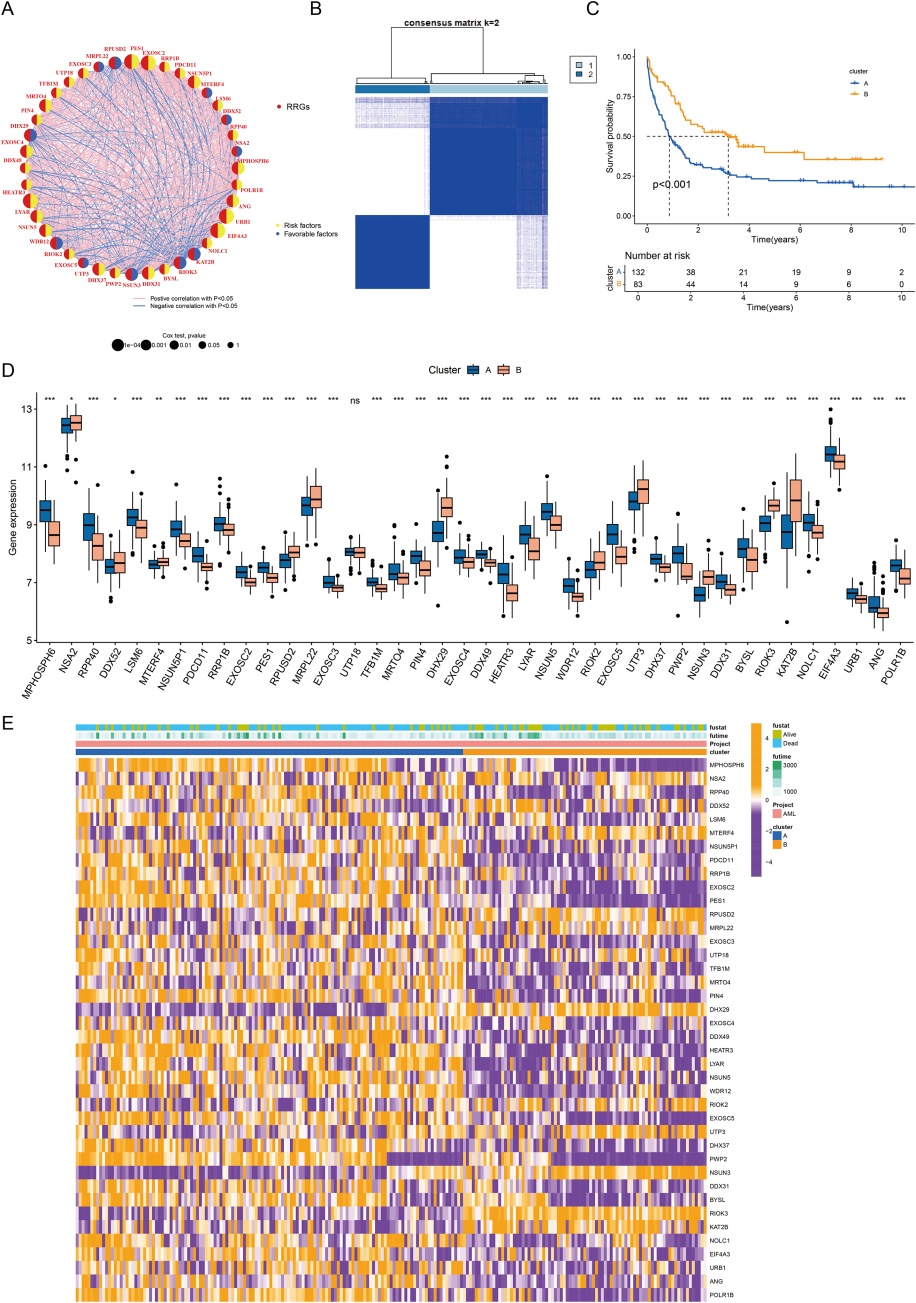
Functional exploration of RRGs. **(A)** Network diagram of univariate Cox regression and correlation analyses for RRGs. **(B)** Consensus clustering using 40 RRGs identified K = 2 as optimal. **(C)** Kaplan-Meier survival analysis comparing the two subtypes. **(D)** Differential gene expression between the two subtypes. **(E)** Heatmap showing RRGs expression across subtypes. * P < 0.05, ** P < 0.01, *** P < 0.001.

**Figure 6:**
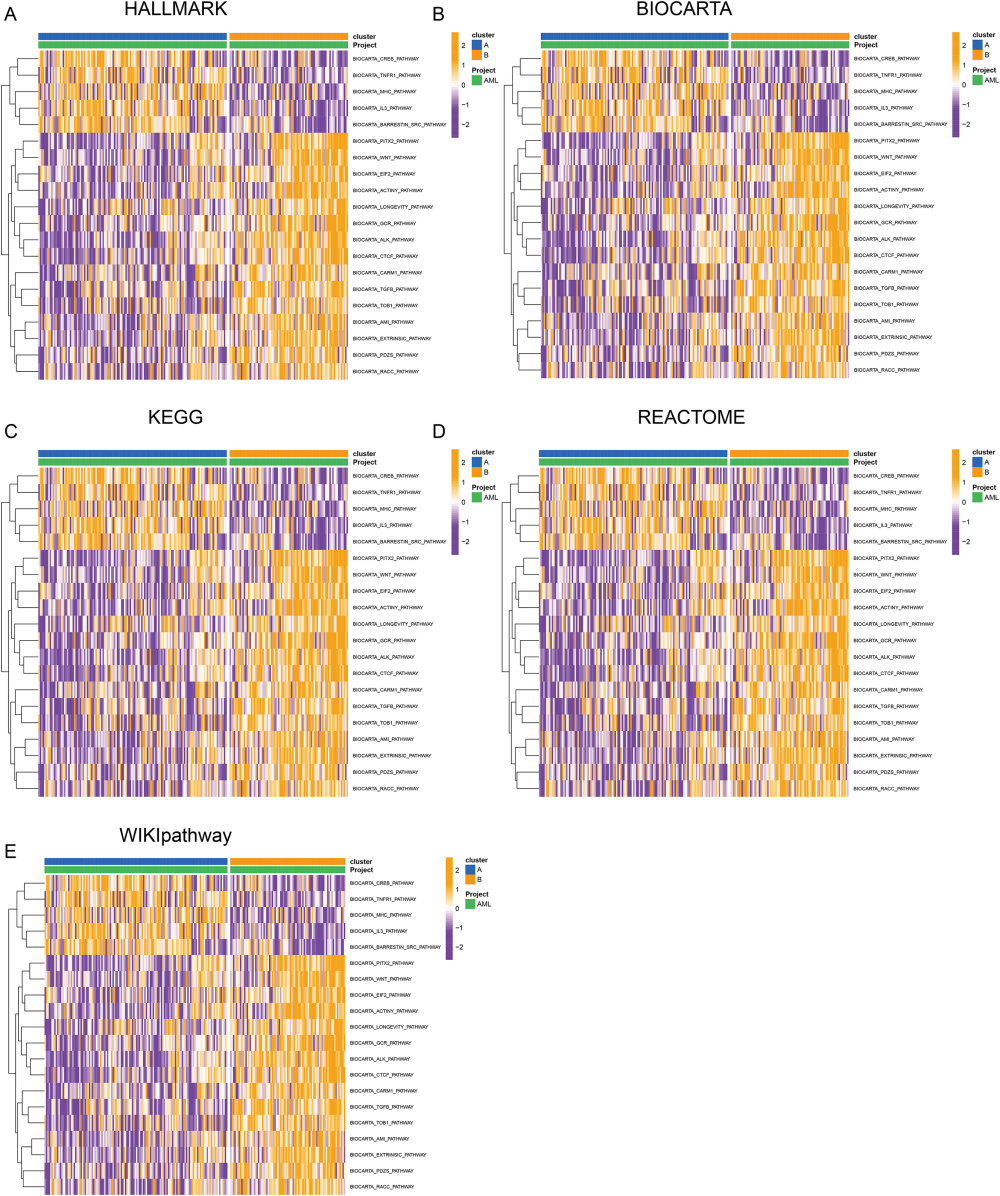
GSVA analysis of key pathways across different subtypes. **(A)** Hallmark pathway enrichment results. **(B)** Biocarta pathway enrichment results. **(C)** KEGG pathway enrichment results. **(D)** Reactome pathway enrichment results. **(E)** Wikipathway enrichment results.

**Figure 7:**
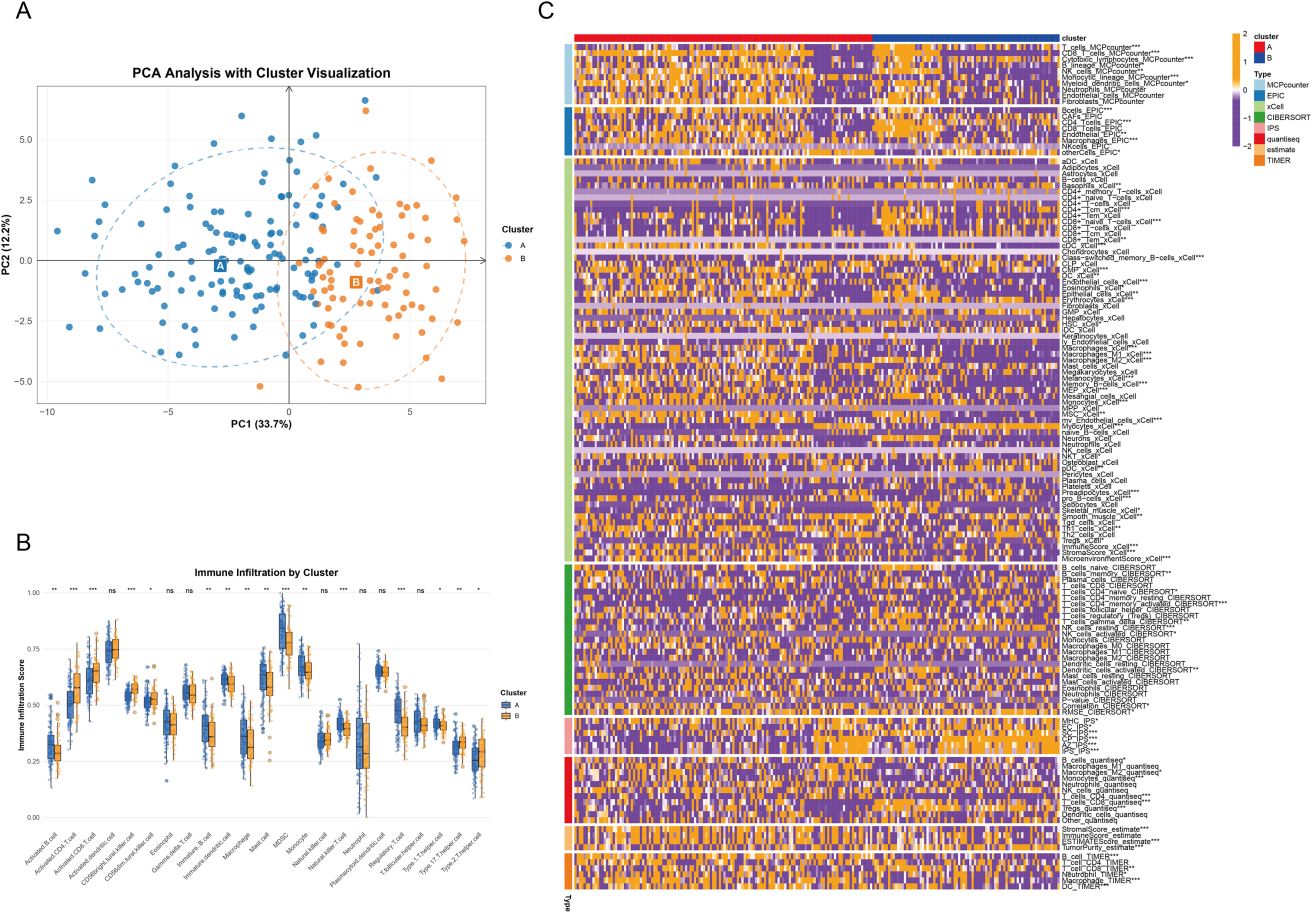
Immune infiltration analysis across different subtypes. **(A)** PCA plot showing the distribution of samples across subtypes. **(B)** Differences in immune cell infiltration between subtypes. **(C)** Heatmap displaying immune cell infiltration across subtypes, analyzed using MCPcounter, EPIC, xCell, CIBERSORT, IPS, quanTIseq, ESTIMATE, and TIMER algorithms.

Univariate Cox regression identified 40 ribosome biogenesis-related genes (RRGs) significantly associated with overall survival (OS) (p < 0.05; **Supplementary Table 5**). Correlation network analysis highlighted EXOSC2, LYAR, and ANG as risk genes and RPUSD2 and RIOK3 as protective genes (**Figure 5A**). Unsupervised clustering defined two stable subtypes (Clusters A/B), with Cluster A exhibiting significantly worse OS (p < 0.001, **Figures 5B, C**) and distinct gene expression patterns (**Figures 5D, E**). GSVA revealed Cluster A enrichment in metabolic reprogramming (fatty acid synthesis, nucleotide metabolism, glycolysis) and mitochondrial stress-immune pathways, while Cluster B was enriched in epigenetic dysregulation (PRC2/H3K27me3), genomic instability, and immunosuppression (**Figures 6A–E**, **Supplementary Table 6**). PCA confirmed transcriptomic separation (**Figure 7A**). ssGSEA showed Cluster A had higher infiltration of immunosuppressive cells (MDSC, macrophages, Tregs; p < 0.05), whereas Cluster B had increased adaptive T cells (CD4^+^/CD8^+^ T, Th17/Th2; p < 0.05) and reduced B cell activity (**Figure 7B**). Integrated immune profiling (IOBR) and ESTIMATE scores indicated Cluster A possesses a stroma/immune-rich microenvironment (higher stromal/combined scores, lower purity; p < 0.01), contrasting with Cluster B’s tumor cell-dominated milieu (higher purity, lower stromal/immune scores; p < 0.001, **Figure 7C**).

### 3.5 Development and validation of the RBS using integrated machine learning

**Figure 8:**
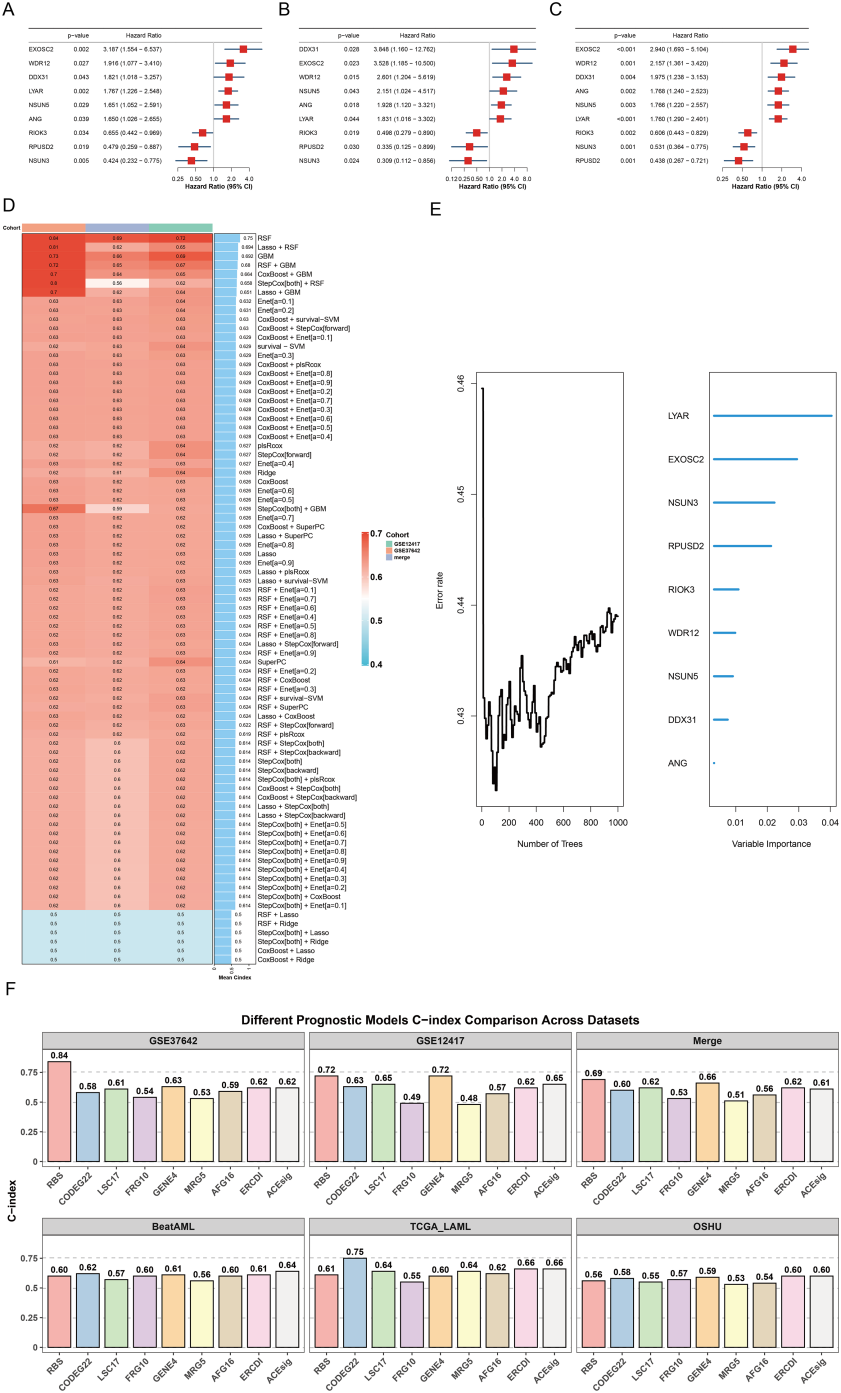
Construction of the RBS model. **(A)** Forest plot of 9 RRGs in GSE37642. **(B)** Forest plot of 9 RRGs in GSE12417. **(C)** Forest plot of 9 RRGs in the merged dataset. **(D)** Among the 81 machine-learning algorithm combinations evaluated across three cohorts, the RSF model demonstrated the highest mean C-index (0.75). **(E)** Error rate and variable importance ranking from the Random Survival Forest (RSF) model. **(F)** C-index analysis comparing the RBS model with eight published risk models across six datasets (GSE37642, GSE12417, Merge, BeatAML, TCGA_LAML, and OHSU).

**Figure 9:**
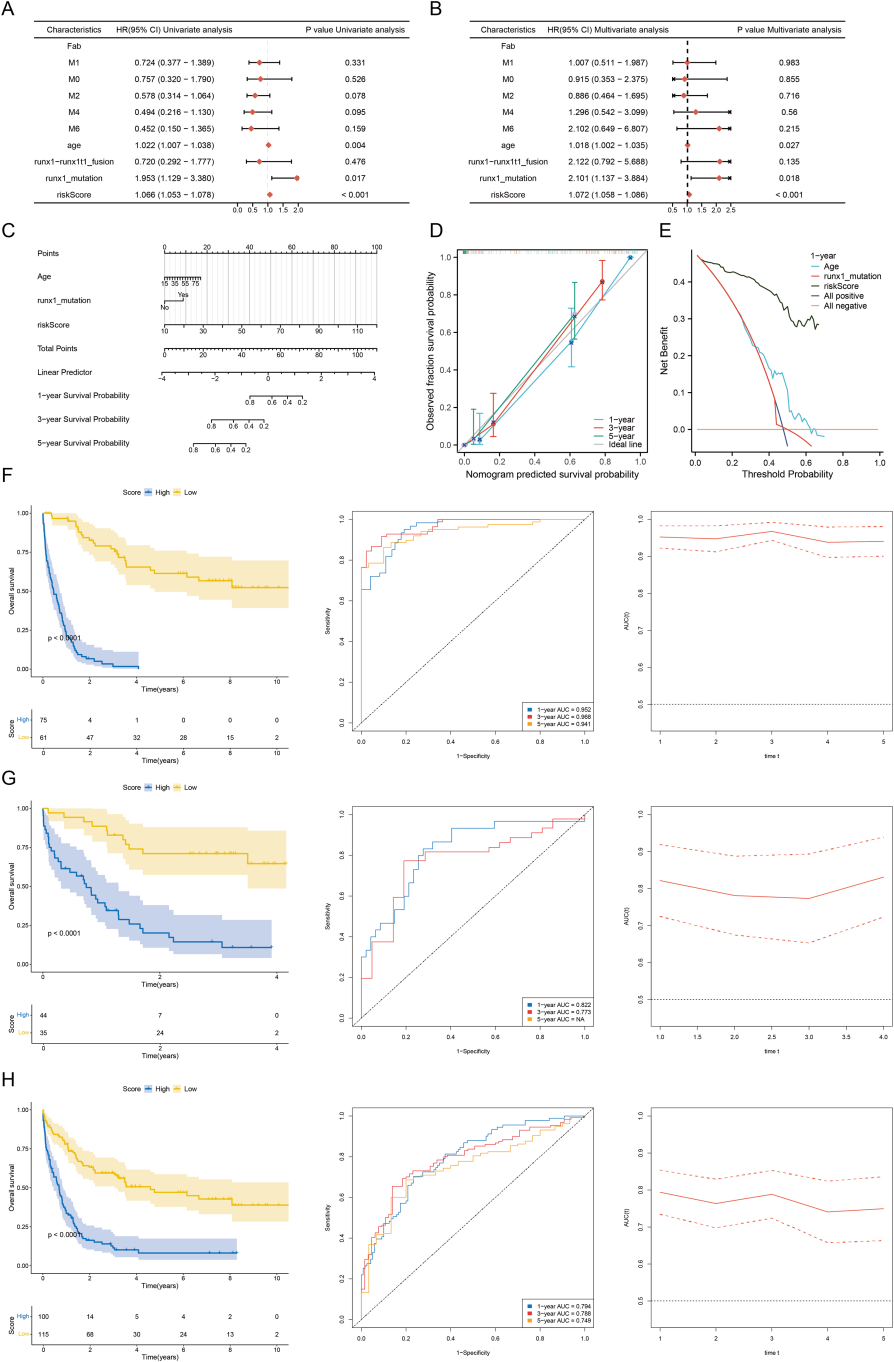
Validation of the RSF model. **(A, B)** Forest plot of independent prognostic predictors identified by univariate **(A)** and multivariate **(B)** Cox regression analyses. **(C–E)** Prognostic Nomogram, Calibration Curve, and Decision Curve Analysis (DCA) in the GSE37642 Dataset. **(F–H)** Survival and time-dependent ROC analyses of high-/low-risk groups across GSE37642, GSE12417, and Merge cohorts.

Univariate Cox regression identified nine prognostic RRGs with consistent hazard ratios across training datasets (**Figures 8A–C**, p < 0.05). Evaluation of 81 machine learning algorithm combinations selected the Random Survival Forest model as optimal (mean C-index = 0.75; **Supplementary Table S7**), achieving minimal error at ~100 trees and ranking the nine key genes (**Figures 8D, E**). Comprehensive validation across six independent cohorts demonstrated the prognostic performance of the RBS model relative to eight established signatures (39–46). In the initial cohorts (GSE37642, GSE12417, and Merge), the RBS model showed superior discriminative ability, achieving the highest C-indices (0.84, 0.72, and 0.69, respectively). In the subsequent independent validation cohorts (BeatAML, TCGA-LAML, and OHSU), the RBS model maintained comparable performance, with C-indices of 0.60, 0.61, and 0.56, placing it within the mid-range of the evaluated signatures. Overall, the RBS model exhibited robust and consistent prognostic capacity across diverse datasets (**Figure 8F**). Multivariate analysis confirmed the RBS-derived riskScore (HR = 1.072, 95% CI 1.058-1.086, p < 0.001), age (HR = 1.018, 95% CI 1.002-1.035, p = 0.027), and RUNX1 mutation (HR = 2.101, 95% CI 1.137-3.884, p = 0.018) as independent predictors after adjusting for clinical covariates including FAB subtype and RUNX1-RUNX1T1 fusion (**Figures 9A, B**). A nomogram integrating riskScore and age demonstrated stable calibration and superior 1-year net clinical benefit (**Figures 9D, E**). Patients stratified by the optimal riskScore cutoff exhibited significantly worse survival in high-risk groups (log-rank p < 0.05), supported by high time-dependent AUCs in GSE37642 (1-year: 0.952, 3-year: 0.968, 5-year: 0.941), GSE12417 (1-year: 0.822, 3-year: 0.773), and the merged cohort (1-year: 0.794, 3-year: 0.788, 5-year: 0.749) (**Figures 9F–H**).

The prognostic value of the RSF model was further validated across three independent cohorts. In the BeatAML cohort, high-risk patients showed significantly shorter overall survival (log-rank p = 0.039), though with modest discriminative ability on time-dependent ROC analysis. Similarly, significant survival stratification was observed in both the TCGA-LAML (p = 0.046) and OHSU (p = 0.038) cohorts. Multivariate analyses incorporating ELN risk category and key mutations consistently identified the riskScore as an independent prognostic factor across all cohorts (**Supplementary Figures S5A–F**).

### 3.6 Feature importance evaluation of the RSF model

**Figure 10:**
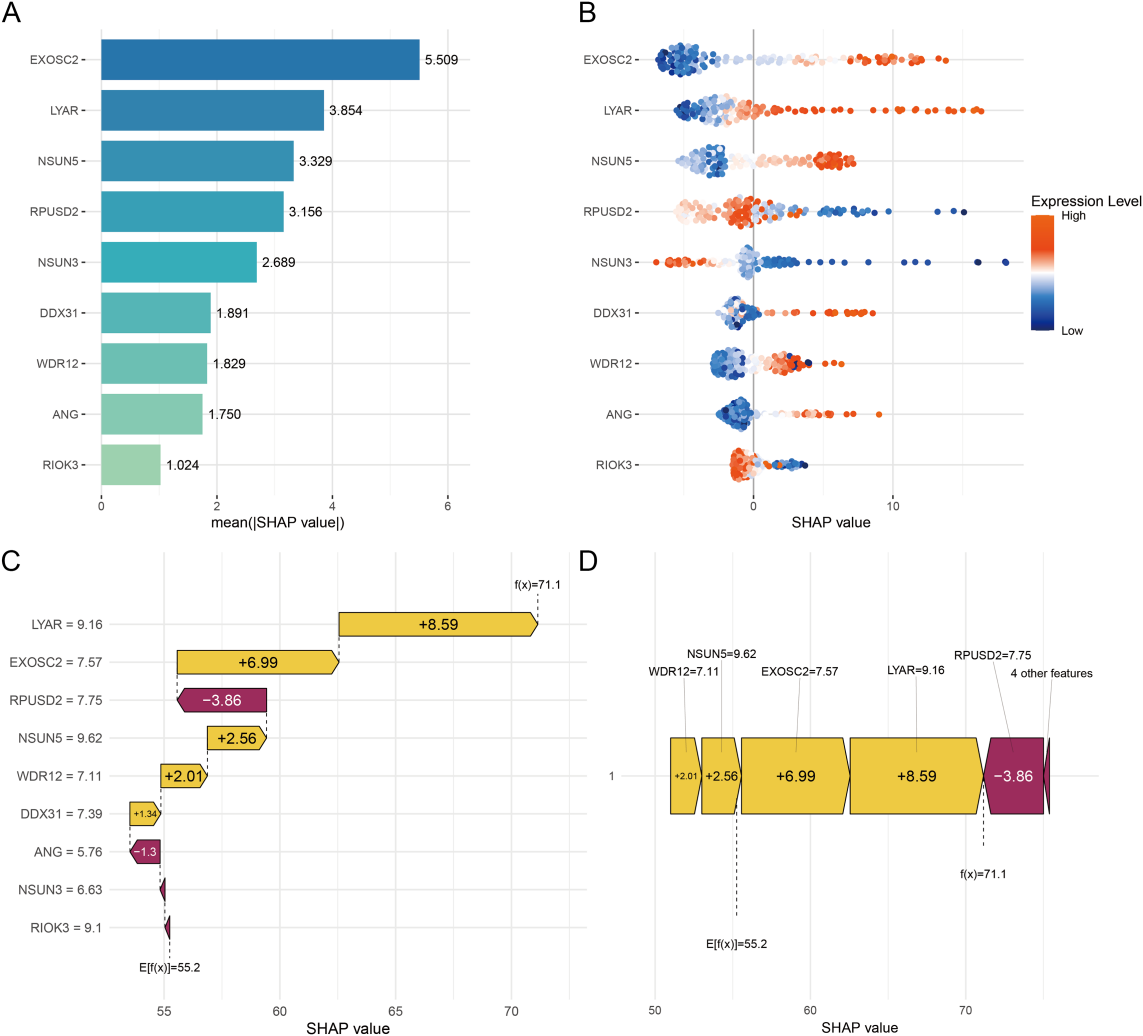
Feature importance evaluation for the RSF model using SHAP methodology. **(A)** SHAP Summary Bar Plot of 9 Feature Genes. **(B)** SHAP beeswarm plot. **(C)** SHAP Waterfall Plot for an Individual Sample. **(D)** SHAP force plot for an Individual Sample.

SHAP-based analysis of the RSF model identified EXOSC2, LYAR, NSUN5, RPUSD2, and NSUN3 as core survival predictors, ranked by mean absolute SHAP values (**Figure 10A**). The SHAP beeswarm plot (**Figure 10B**) revealed high expression of EXOSC2, LYAR, and NSUN5 correlated with increased mortality risk, whereas elevated RPUSD2 and NSUN3 expression associated with prolonged survival; gene expression levels (color-coded) and SHAP values (horizontal distribution) demonstrated risk-increasing genes (e.g., EXOSC2) elevated risk scores via positive SHAP values, while protective genes (e.g., RPUSD2) reduced scores via negative values, with effects amplified at higher expression. Individual sample analysis (**Figures 10C, D**) indicated high EXOSC2 (+6.99) and LYAR (+8.59) expression significantly increased mortality risk, whereas elevated RPUSD2 (-3.86) exerted protective effects; the net SHAP sum (+19.49 from risk genes and -5.16 from protective genes), combined with the baseline score (55.2), yielded a final patient risk score of 71.1. SHAP analysis across five independent cohorts consistently identified EXOSC2 and LYAR as the dominant predictors, with minor ranking variations reflecting cohort heterogeneity but overall supporting RBS signature robustness (**Supplementary Figures S6A–E**).

### 3.7 Underlying biological mechanisms associated with RBS

RBS correlated significantly with multiple genes. Heatmaps of the top 50 positively/negatively correlated genes (Pearson coefficients) revealed distinct risk-group-specific expression patterns (**Supplementary Figures S7A, B**). Gene set enrichment analysis indicated high-RBS samples exhibited metabolic-proliferation synergy dysregulation: enhanced mitochondrial energy metabolism (oxidative phosphorylation, TCA cycle) and respiratory chain activity supporting malignant proliferation; concurrent activation of nucleotide synthesis and translational pathways (e.g., tRNA aminoacylation) meeting anabolic demands; and reinforced DNA repair (homologous recombination, nucleotide excision repair) and telomere homeostasis contributing to adverse prognosis. Conversely, low-RBS samples were enriched in homeostasis-immune regulatory networks: autophagy-mediated mitochondrial quality control cleared dysfunctional components with FoxO signaling enhancing stress resistance; MHC class I antigen presentation-ubiquitination systems synergistically enhanced immune surveillance; and polycomb complexes suppressed aberrant transcription via epigenetic silencing, collectively inhibiting proliferation and improving prognosis (**Supplementary Figures S7C–E**).

### 3.8 Analysis of immune microenvironment, cytokines, and immunotherapy response

**Figure 11:**
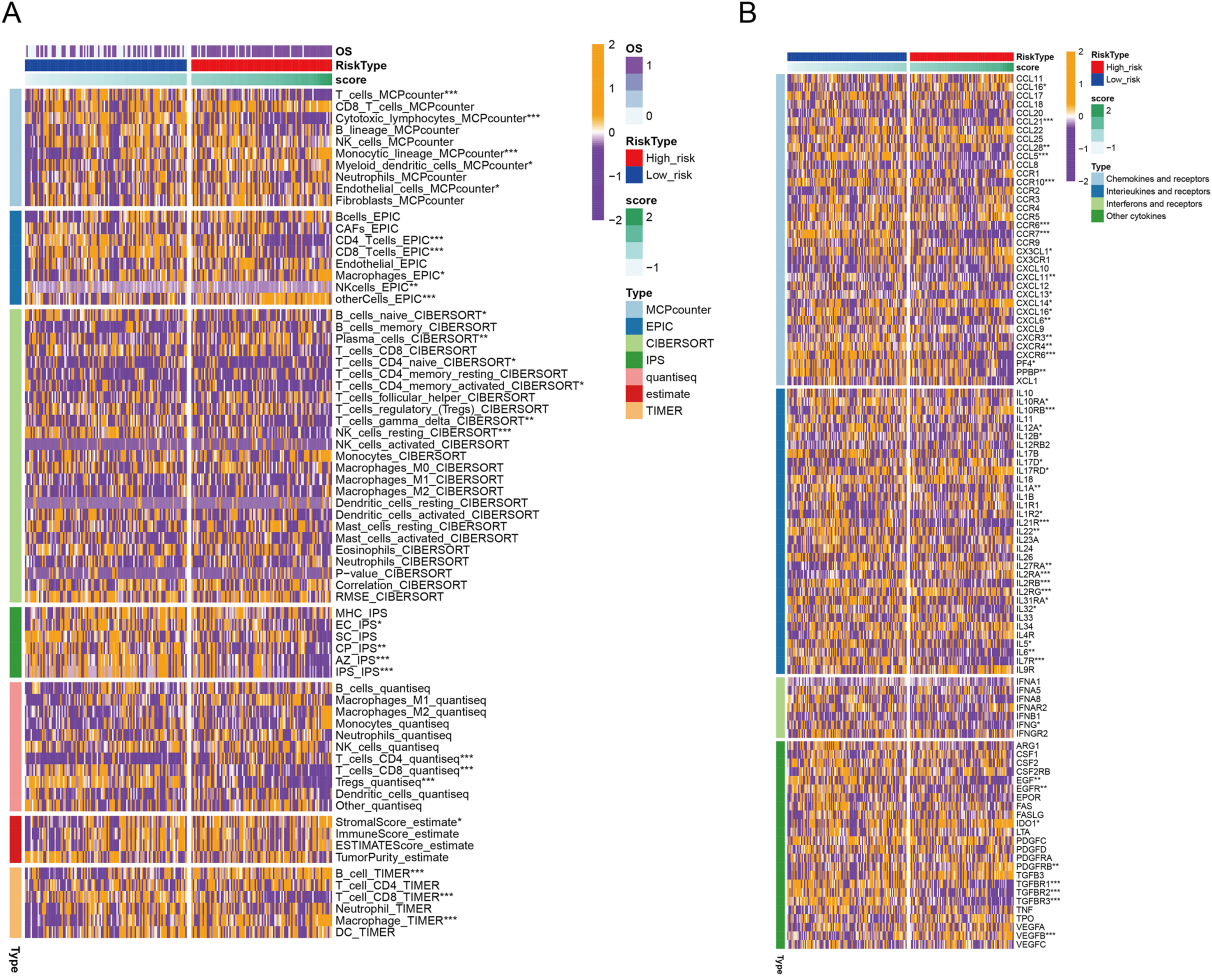
Immunocyte infiltration and chemokine-receptor profiling stratified by RBS group **(A)** Immune Infiltration Analysis in High- vs Low-RBS Groups. **(B)** Comprehensive Cytokine-Receptor Profiling in High- vs Low-RBS Groups.

**Figure 12:**
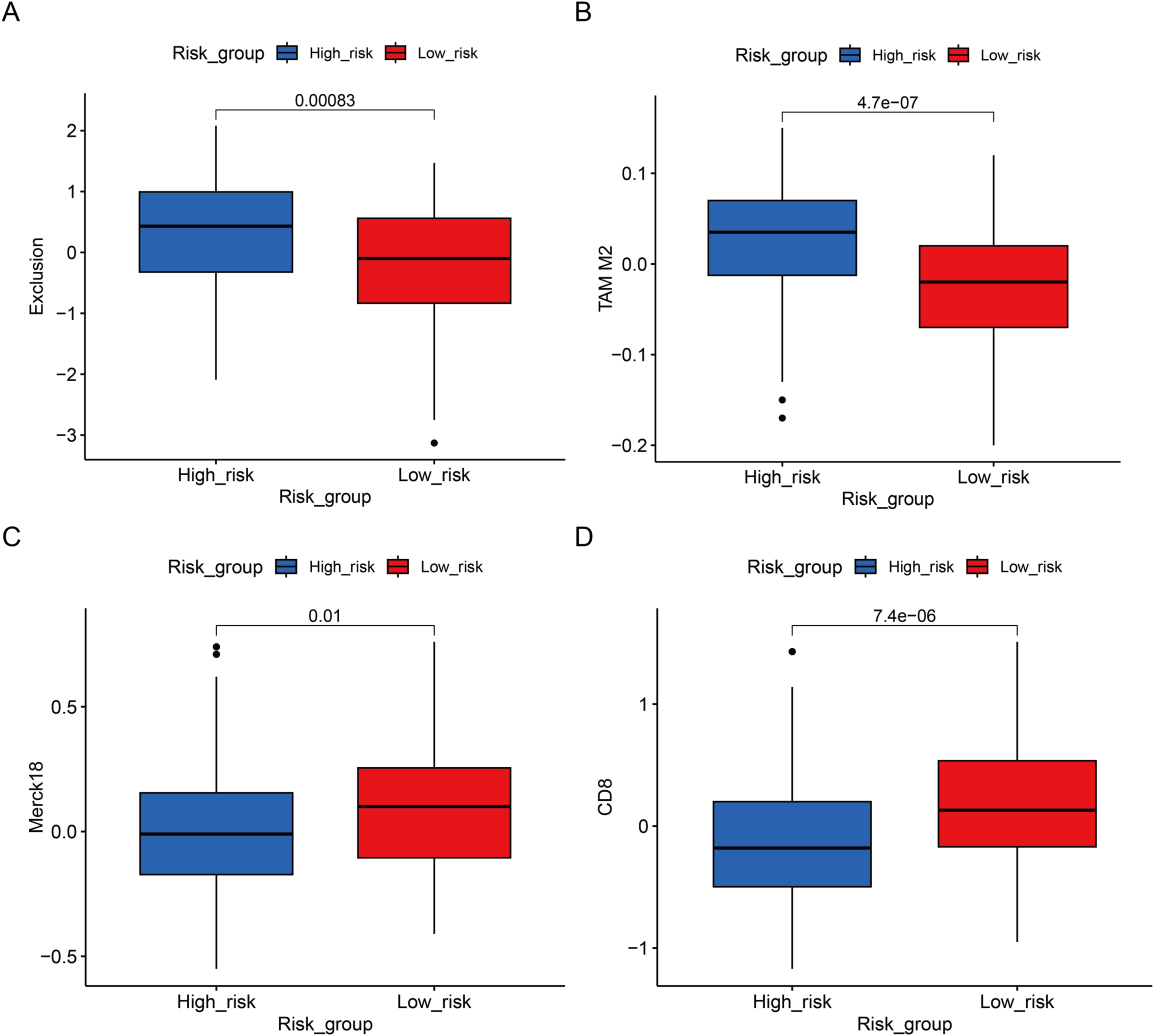
Immune response profiles in high- and low-RBS groups. **(A)** Differences in Exclusion Scores Between High- and Low-RBS Groups. **(B)** Differences in TAM M2 Scores Between High- and Low-RBS Groups. **(C)** Differences in Merck18 Scores Between High- and Low-RBS Groups. **(D)** Differences in CD8 Scores Between High- and Low-RBS Groups.

High-RBS samples exhibited elevated stromal scores and enrichment in immunosuppressive components (M2 macrophages, Tregs, IDO1), with reduced cytotoxic lymphocytes (CD8^+^ T/NK cells) (**Figure 11A**, **Supplementary Table 8**). Cytokine profiling revealed upregulation of immune-recruiting chemokines (CCL16, CX3CL1), pro-angiogenic factors (VEGFB, CXCL14), and immunosuppressive receptors (IL10RB, IL1R2) alongside downregulated pro-inflammatory cytokines (IL12A/B, IFN-γ) in high-RBS group; conversely, low-RBS samples showed elevated immune-activating chemokines (CCL5, CXCL13), IFN-γ (potentiating NK cells), and immunomodulatory TGFBR (**Figure 11B**, **Supplementary Table 9**). Thus, high-RBS tumors displayed an immunosuppressive, angiogenesis-prone microenvironment with impaired effector immunity, correlating with poor prognosis. In immunotherapy contexts, the high-RBS group demonstrated increased immune exclusion scores and TAM_M2 infiltration but decreased CD8^+^ T-cell markers and Merck18 expression, indicating T-cell exclusion, immunosuppression, and functional impairment; low-RBS tumors exhibited enhanced T-cell infiltration and immunogenic activity, supporting superior immunotherapy response potential (**Figures 12A–D**, **Supplementary Table 10**). High-RBS resistance may stem from M2 macrophage-mediated suppression and physical barriers, while low-RBS tumors benefit from enhanced T-cell recruitment.

### 3.9 Drug sensitivity analysis and molecular docking

**Figure 13:**
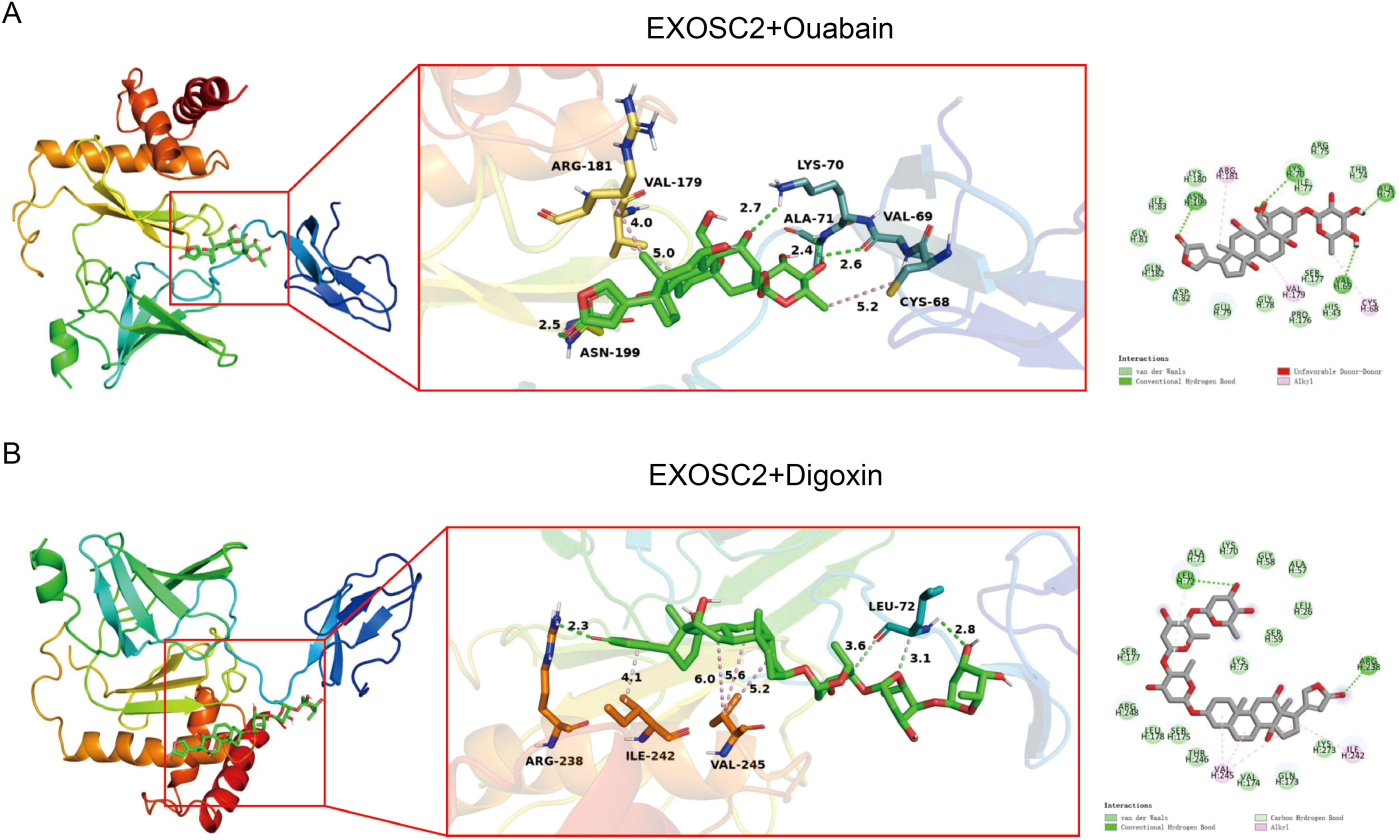
Molecular docking. **(A)** Interaction Between EXOSC2 and Ouabain. **(B)** Interaction Between EXOSC2 and Digoxin.

Drug sensitivity profiling revealed high-risk AML patients exhibited increased vulnerability to inhibitors targeting key pathways: cell cycle regulators (e.g., CDK4/6 inhibitors), apoptosis inducers (e.g., BCL-2 inhibitors), and epigenetic modulators (e.g., HDAC inhibitors), with additional efficacy against DNA damage repair and tyrosine kinase pathways, suggesting dependencies on cell cycle dysregulation, apoptosis resistance, and epigenetic remodeling (**Supplementary Figure S8**, **Supplementary Table 11**). Computational screening of the top 4 RSF-selected prognostic genes identified EXOSC2 – significantly overexpressed in AML – as strongly associated with ouabain (P = 0.009, OR = 106.87) and digoxin (P = 0.051, OR = 43.44). Molecular docking confirmed strong binding of both Na^+^/K^+^-ATPase inhibitors to EXOSC2, with binding energies of -8.3 kcal/mol (ouabain, **Figure 13A**) and -8.8 kcal/mol (digoxin, **Figure 13B**), exceeding the -8 kcal/mol threshold for high-affinity interactions, supporting their potential anti-leukemic efficacy via EXOSC2 targeting.

### 3.10 qRT-PCR and western blot validation of key RRGs

**Figure 14:**
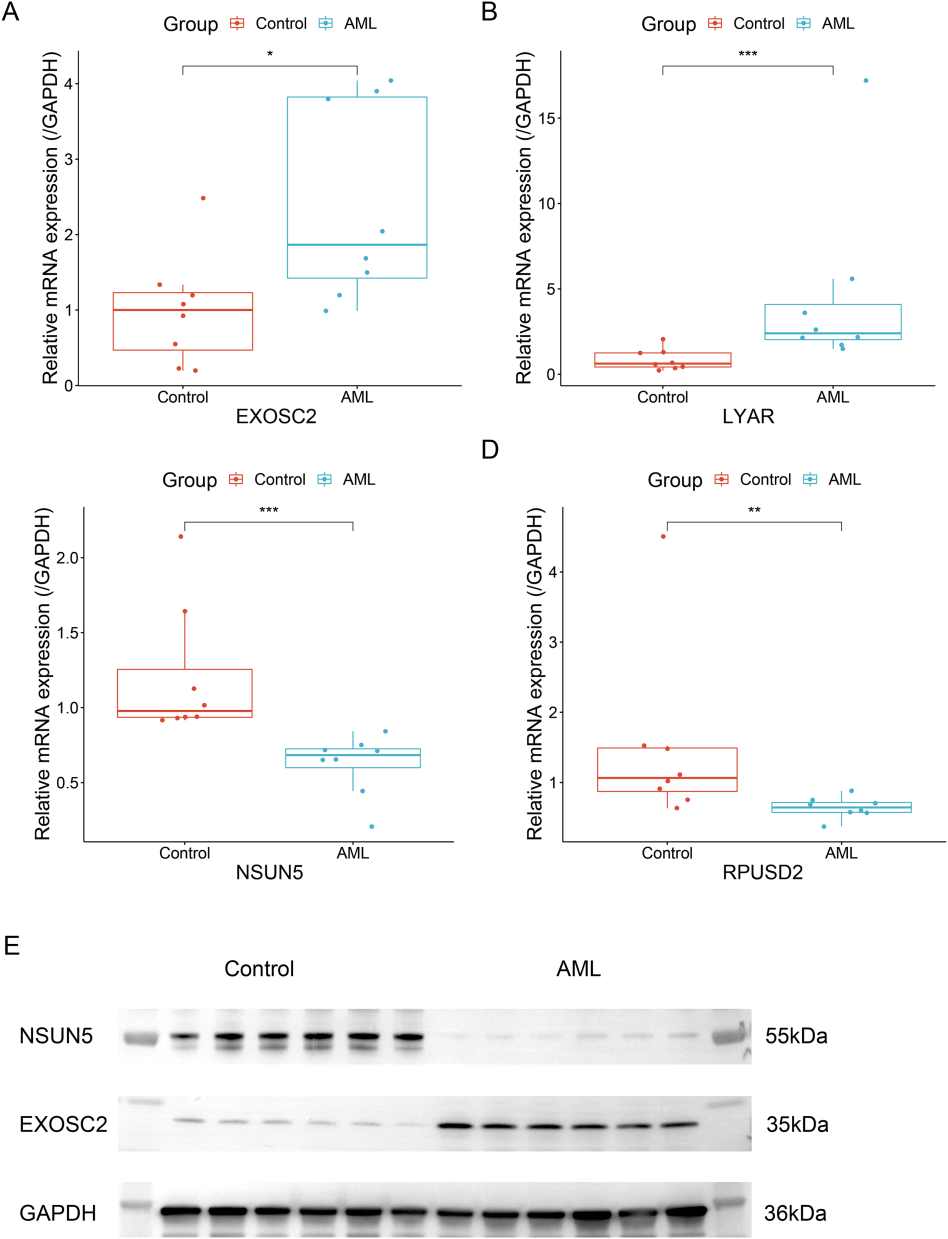
Validation of key gene expression by RT-qPCR and western blotting **(A)** EXOSC2 mRNA expression analyzed by RT-qPCR. **(B)** LYAR mRNA expression analyzed by RT-qPCR. **(C)** NSUN5 mRNA expression analyzed by RT-qPCR. **(D)** RPUSD2 mRNA expression analyzed by RT-qPCR. **(E)** Protein expression of NSUN5 and EXOSC2 detected by Western blotting (GAPDH: loading control). * P < 0.05, ** P < 0.01, *** P < 0.001.

In a preliminary qRT-PCR analysis of four top-ranked RRGs using our limited sample set, we observed trends of elevated mRNA expression for EXOSC2 and LYAR, alongside decreased levels of NSUN5 and RPUSD2 in AML patients compared to controls (**Figures 14A–D**). Although SHAP analysis indicated NSUN5 high expression was prognostically detrimental, its observed mRNA downregulation in our cohort may suggest potential post-transcriptional regulation or sample heterogeneity. The expression trends of the other three genes were consistent with their SHAP-predicted pathogenic roles. At the protein level, Western blot analysis provided supportive evidence, showing upregulation of EXOSC2 and downregulation of NSUN5 in AML samples (**Figure 14E**), which aligned with the transcriptional trends.

## 4 Discussion

The global disease burden of AML continues to increase, with rising age-standardized incidence rates (ASIR) posing a growing public health challenge (47, 48). We recognize the urgent need to identify biomarkers guiding precision diagnosis and treatment. Recent advances in scRNA-seq empower deep exploration of tumor cellular heterogeneity, construction of cross-species atlases, identification of critical subpopulations, and elucidation of their functional roles within the TME (49). Dysregulation of RiboSis acts as a key driver during tumor evolution. Published evidence confirms its dysfunction closely associates with cancer initiation, progression, metastasis, and drug resistance, establishing RiboSis as a highly promising anti-cancer therapeutic target (50–52). Nevertheless, the biomarker potential of RRGs in AML remains underexplored, and their application value—including single-cell expression characteristics, prognostic modeling, and targeted intervention prediction—requires further investigation.

Our single-cell transcriptomic analysis mapped the dynamic regulatory landscape of RiboSis in AML, providing a cellular resolution perspective that moves beyond bulk-level analyses. We revealed abnormally elevated RiboSis activity specifically within hematopoietic stem/progenitor cell (HSCs/GMPs/MEPs) subpopulations. These cells displayed significantly higher RRG expression than terminally differentiated cells, concurrently showing increased genomic instability and global metabolic reprogramming. This remodeling manifested as coordinated enhancement of glycolysis and oxidative phosphorylation alongside activation of purine/pyrimidine nucleotide biosynthesis, collectively supplying energy and biosynthetic substrates for malignant clonal expansion. We further identified that high-RiboSis-activity subpopulations drive microenvironmental immunosuppression via the MIF-(CD74+CD44) axis: CellChat analysis validated this axis promotes myeloid cell polarization toward M2-type tumor-associated macrophages while impairing T-cell activation. This constitutes a previously underappreciated mechanism linking ribosome biogenesis to immune evasion in AML. Functional enrichment also revealed potential drug resistance mechanisms—upregulated glutathione metabolism enhances reactive oxygen species (ROS) scavenging, and cytochrome P450-mediated drug metabolism pathway activation potentially facilitates chemotherapeutic agent degradation. Together, RiboSis dysregulation disrupts energy metabolism, biosynthesis, and immune functionality, thereby accelerating AML pathogenesis.

Based on RRG consensus clustering, we defined two AML subtypes with significant prognostic differences: Cluster A showed metabolic-immune co-dysregulation, while Cluster B maintained adaptive immune responses. To transcend the limitations of single-gene biomarkers, we integrated multiple machine learning algorithms to develop a composite ribosome biogenesis signature (RBS), with the Random Survival Forest (RSF) algorithm achieving optimal predictive performance. The RBS model produced risk scores based on nine core RRGs (EXOSC2, LYAR, NSUN5, RPUSD2, NSUN3, RIOK3, ANG, RRP7A, RPUSD1), confirmed as independent prognostic factors in multivariate analysis. SHAP interpretability analysis identified EXOSC2, LYAR, and NSUN5 as key risk drivers, while RPUSD2 and NSUN3 conferred protective effects; SHAP dependency plots illustrated dose-response relationships between gene expression and risk contributions. The model sustained high predictive accuracy across independent cohorts and substantially improved individualized prognostic assessment through a clinically integrated nomogram.

LYAR (human Ly-1 antibody reactive homolog), a nucleolar protein containing zinc-finger DNA-binding domains and nuclear localization signals, demonstrates high expression in testis where it regulates cell proliferation and RNA metabolism (53). Existing studies document its cancer-promoting roles: in colorectal cancer it mediates metastasis via the FSCN1/fatty acid metabolism axis (54); in hepatocellular carcinoma it drives proliferation/migration and correlates with advanced staging and vascular invasion (55); in non-small cell lung cancer it promotes proliferation through the Cyclin A/PCNA pathway, leading to poor prognosis (56)—though its function in AML lacks reports. RPUSD2, an RNA-modifying pseudouridine synthase, has sparse research linking it to colorectal cancer metastasis and hepatocellular carcinoma (57, 58). NSUN3 serves as a mitochondrial-specific methyltransferase that preserves mitochondrial translation and energy metabolism by catalyzing critical tRNA modifications (59, 60). Its dysregulation triggers multisystem disorders: mutations induce mitochondrial encephalomyopathy and optic neuropathy (61, 62); it exacerbates inflammatory responses in sepsis-induced kidney injury (63); and impairs respiratory chain function in hemoglobin H disease (64). Importantly, during leukemogenesis, NSUN3 adopts extra-mitochondrial functions—through cooperative assembly of 5-AZA-sensitive chromatin complexes with hnRNPK, it modulates recruitment of lineage transcription factors (GATA1/SPI1) and RNA polymerase II activity, thereby governing therapeutic drug responsiveness (65).

Our analyses provide further support that ribosomal dysregulation may contribute to an immunosuppressive microenvironment in AML. This aligns with the emerging view from ribosomopathies, which posits that ribosomal dysfunction can promote leukemogenesis by impairing immune surveillance. Experimental validation confirmed the robust prognostic value of the RBS in AML. The high-RBS group presented an immunosuppressive microenvironment characterized by increased myeloid-derived suppressor cell infiltration, CD8^+^ T-cell exhaustion, and enhanced immune exclusion, collectively linking to diminished immunotherapy response and poor outcomes. Conversely, the low-RBS group exhibited immune-activated states marked by effector T-cell expansion and upregulated chemokines CCL5/CXCL13, forming a therapeutically favorable niche. Mechanistically, our validation focused on core genes EXOSC2 and NSUN5: qRT-PCR and Western blot validated significant EXOSC2 overexpression in AML (aligning with its SHAP risk contribution), while NSUN5—despite being a risk factor—showed paradoxical downregulation, suggesting potential post-transcriptional regulation. Intriguingly, molecular docking disclosed high-affinity interactions between EXOSC2 and cardiac glycosides (ouabain/digoxin, binding energy < -8 kcal/mol). Given the documented yet mechanistically incompletely understood anti-leukemic effects of these compounds (35), this finding suggests a novel and testable hypothesis: that targeting EXOSC2 may represent a previously unrecognized mechanism contributing to their efficacy in AML. Functionally, EXOSC2 acts as a core structural component of the RNA exosome complex (S1/KH domain family), critical for 3’-end RNA processing/degradation and RNA homeostasis maintenance (66), whereas NSUN5—a cytosine-5 RNA methyltransferase—primarily catalyzes m^5^C RNA modifications for post-transcriptional regulation (67). Thus, our study pioneers the functional characterization of these RRGs and delivers a multi-faceted prognostic tool, while also revealing EXOSC2 as a novel, therapeutically actionable target.

The RBS shows significant association with AML patient prognosis, displaying exceptional predictive power and immune microenvironment relevance that underscore its potential as a clinical biomarker. With advances in AML immunotherapy, RBS effectively stratifies patient response heterogeneity; integrating RBS into clinical practice could advance personalized risk stratification and immunotherapy decision-making, potentially improving survival outcomes.

We acknowledge several limitations in our study. First, the retrospective nature of our analysis, reliant on public databases, may introduce batch effects and incomplete clinical annotations. Second, while our single-cell analysis and sample-level cell communication approach captured dominant ecosystem patterns, it may undersample rare intercellular interactions and necessitates future spatial transcriptomics for precise niche characterization. Third, our mechanistic inferences regarding key genes like EXOSC2—particularly their roles in immunosuppression or drug resistance—remain primarily association-based and supported by computational docking; these findings are thus hypothesis-generating and require direct functional validation through gene perturbation and detailed mechanistic studies in the future. Finally, the experimental validation was constrained by the cohort scale and depth, which limits the robustness of the clinical correlations and leaves the functional networks of RRGs incompletely elucidated. Future work should involve multi-center prospective cohorts and functional genomics to definitively confirm the clinical relevance of core genes like EXOSC2/NSUN5 and decipher their downstream pathways.

## 5 Conclusion

This study investigates the critical role of RiboSis in AML progression through single-cell transcriptomics. We found that malignant progenitors (HSCs/GMPs/MEPs) display specifically elevated RiboSis activity, strongly linked to genomic instability, metabolic reprogramming, and an MIF-CD74-induced immunosuppressive microenvironment. We established a multi-algorithm-integrated RBS prognostic model and applied the SHAP framework to clarify the prognostic contributions of its feature genes, bridging predictive performance with biological mechanisms. The RBS stratifies patients into immune-activated (low-RBS) and immune-suppressed (high-RBS) subtypes, revealing significant differences in tumor microenvironment, immunotherapy response, and clinical outcomes. Experimental validation verified the pathological dysregulation of core genes. Critically, molecular docking predicted high-affinity binding of ouabain/digoxin to EXOSC2 (binding energy < -8 kcal/mol), demonstrating its targetability and proposing novel clinical translation avenues. Collectively, our work positions ribosome biogenesis as a central regulator that links malignant transformation to immune evasion in AML. These results offer essential theoretical foundations and practical guidance for precise AML prognosis stratification, immunotherapy beneficiary selection, and personalized targeting strategy development.

## Data Availability

The original contributions presented in the study are included in the article/**Supplementary Material**. Further inquiries can be directed to the corresponding author.
