# Supplementary material for: Elucidating AML ribosome biogenesis reshaping prognostic assessment and immune microenvironment integrated single-cell and bulk RNA analysis
Source: Front Immunol. 2025 Nov 28;16:1644671. doi: 10.3389/fimmu.2025.1644671 (PMC12698545; doi:10.3389/fimmu.2025.1644671)
Supplement: Supplementary file 1 [file Table1.docx]

**
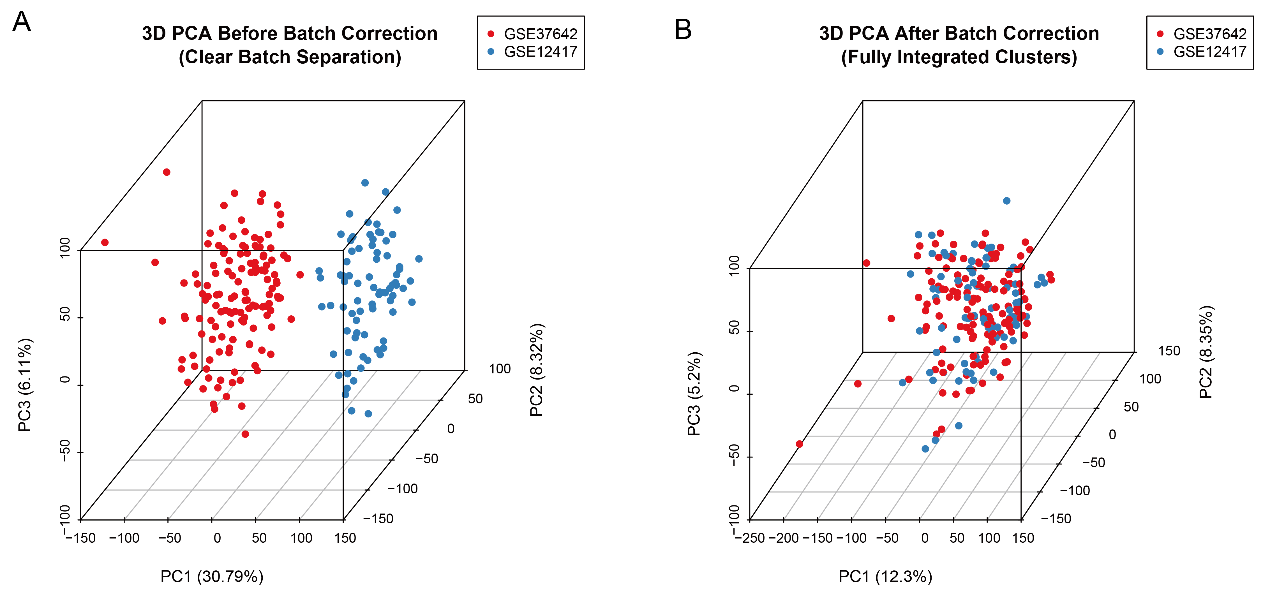
**

**Supplementary Figure S1. 3D PCA Plots Before and After Batch Correction**

A. 3D PCA Before Batch Correction (Clear Batch Separation)

Red dots: GSE37642; Blue dots: GSE12417. PC1: 30.79%, PC2: 8.32%, PC3: 6.11%.

B. 3D PCA After Batch Correction (Fully Integrated Clusters)

Red dots: GSE37642; Blue dots: GSE12417. PC1: 12.3%, PC2: 8.35%, PC3: 5.2%.

**
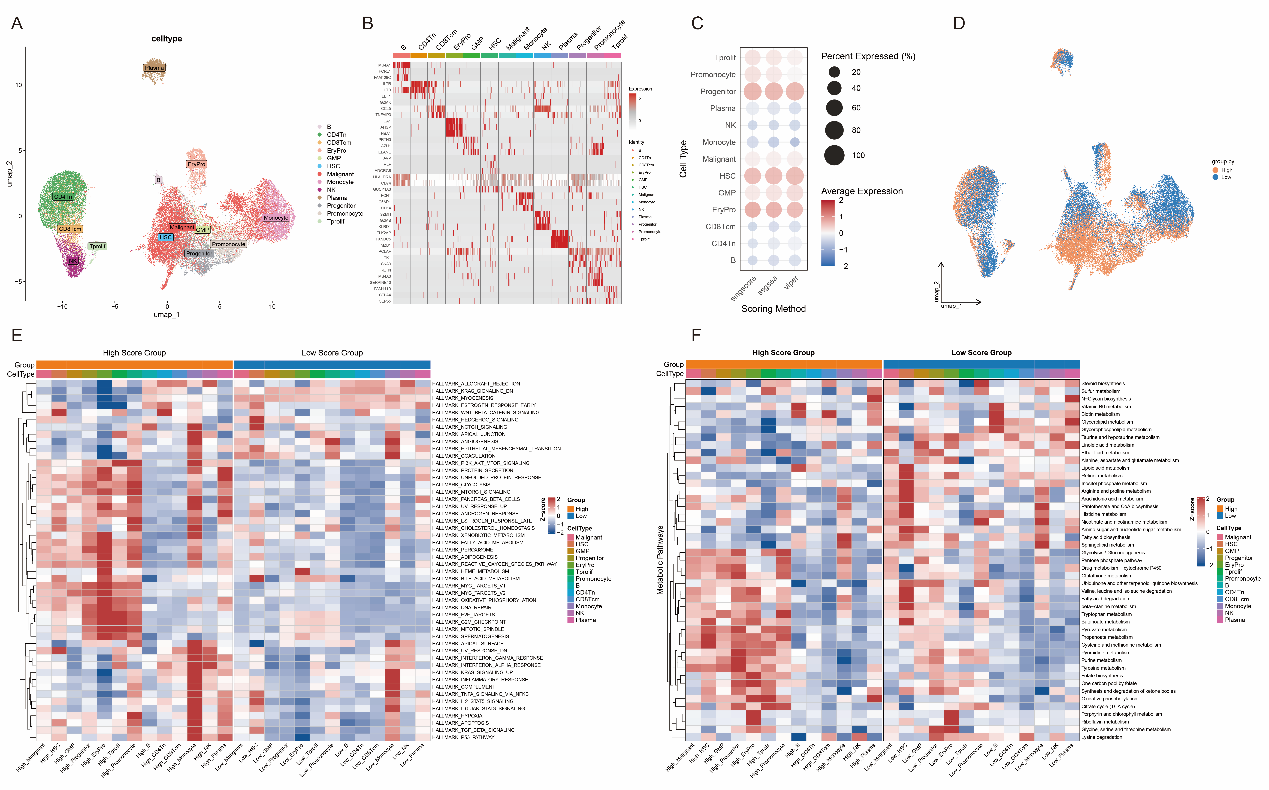
**

**Supplementary Figure S2. Integrated Analysis of AML scRNA-seq Data (GSE116256)**

(A) The UMAP visualization depicts 13 annotated cell types.

(B) Heatmap showing the top 3 marker genes for each cell type.

(C) Bubble plot comparing RiboSis activity scores across 13 cell subtypes using three algorithms.

(D) The UMAP plot displays cell distribution in high and low RiboSis activity groups.

(E) The heatmap shows HALLMARK pathway scores (z-scores) across cell subpopulations in different RiboSis activity groups (red: high scores, blue: low scores).

(F) Metabolic pathway scores across cell subpopulations by RiboSis activity group.

**
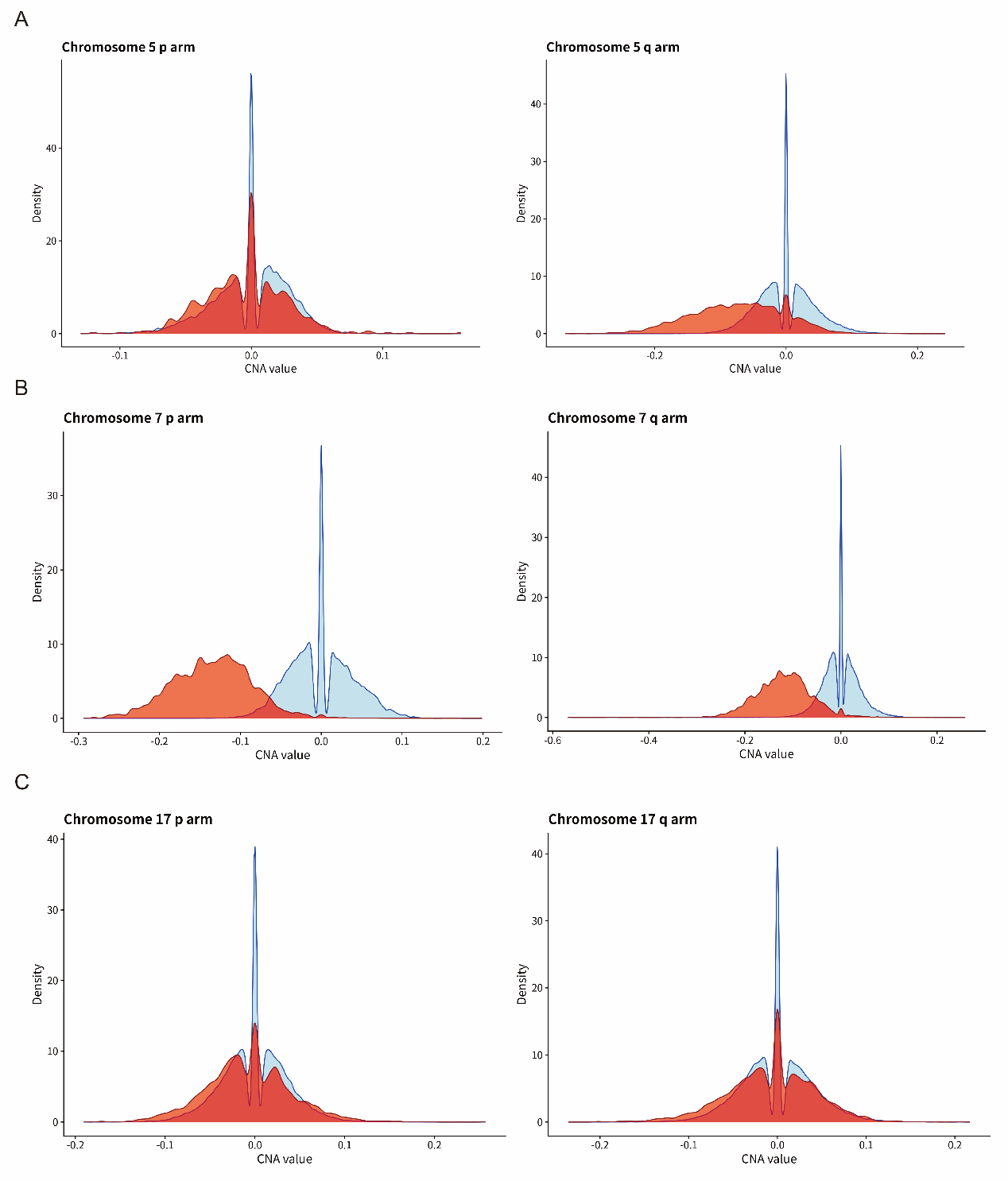
**

**Supplementary Figure S3. Chromosomal copy number alteration (CNA) analysis by copyKAT.** Density distribution plots of copy number alterations for specific chromosome arms. The x-axis represents the "CNA value", indicating the relative copy number level. The y-axis represents the "Density", indicating the frequency distribution of cells.

(A) Density distribution of CNAs for the short arm (5p) and long arm (5q) of chromosome 5.

(B) Density distribution of CNAs for the short arm (7p) and long arm (7q) of chromosome 7.

(C) Density distribution of CNAs for the short arm (17p) and long arm (17q) of chromosome 17.

**
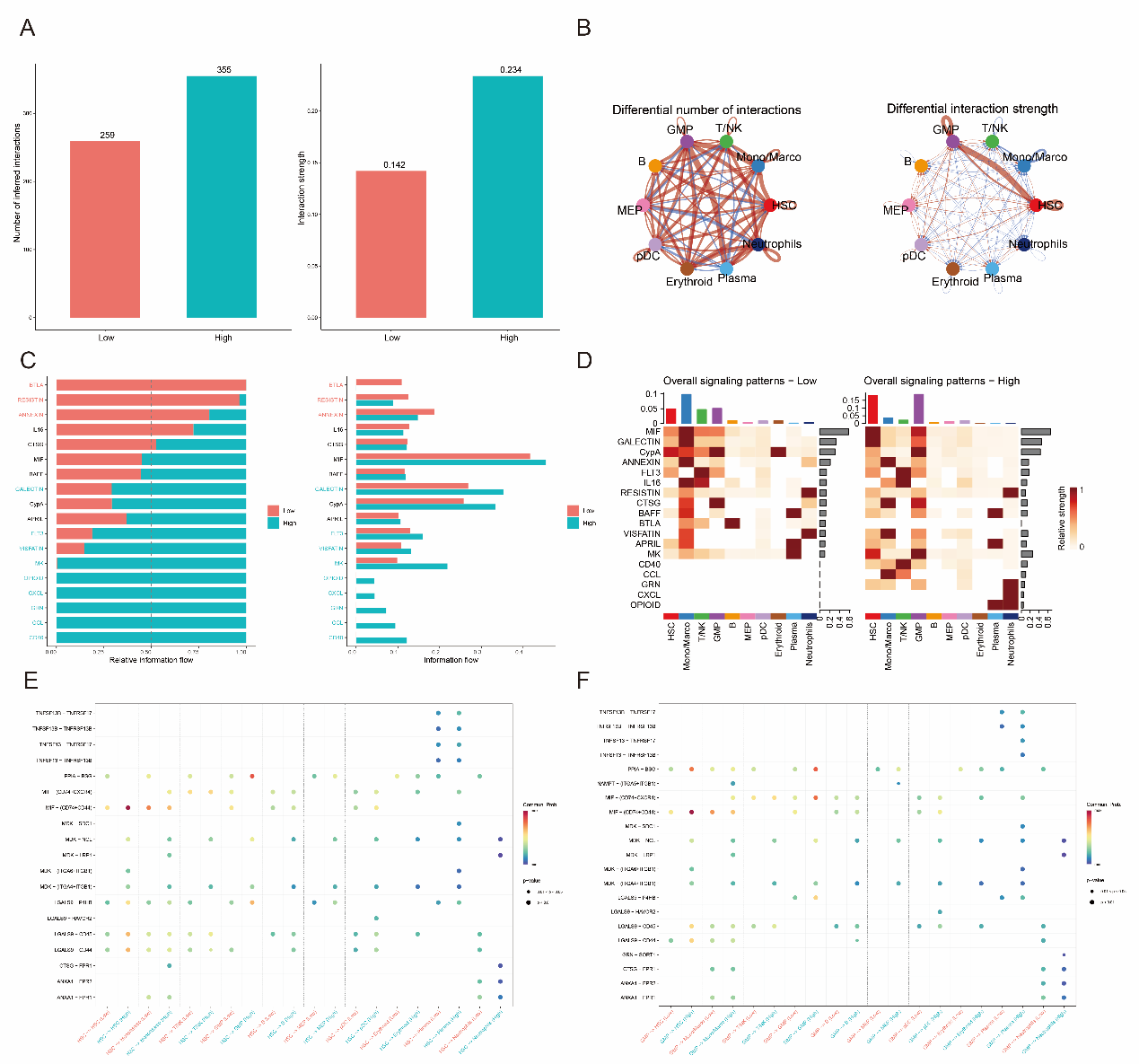
**

**Supplementary Figure S4. Comparative analysis of cell-cell interactions between high and low RiboSis activity groups.**

(A) The bar chart shows the total number (left) and weight (right) of ligand-receptor interactions between high- and low-RAS groups.

(B) Differential communication network showing overall quantity and strength differences. Red lines: stronger in high-RAS group; blue lines: stronger in low-RAS group.

(C) Comparison of pathway signaling activity indicating group-specific pathway enhancements in high- and low-RAS groups.

(D) Heatmap depicting overall pathway signaling activity across high- and low-RAS groups, with pathways in rows and cell subsets in columns. Deepening color indicates increasing signaling strength.

(E) Dot plot displays signaling activity changes in HSC relative to other cell types.

(F) Dot plot displays signaling activity changes in GMP relative to other cell types.

**
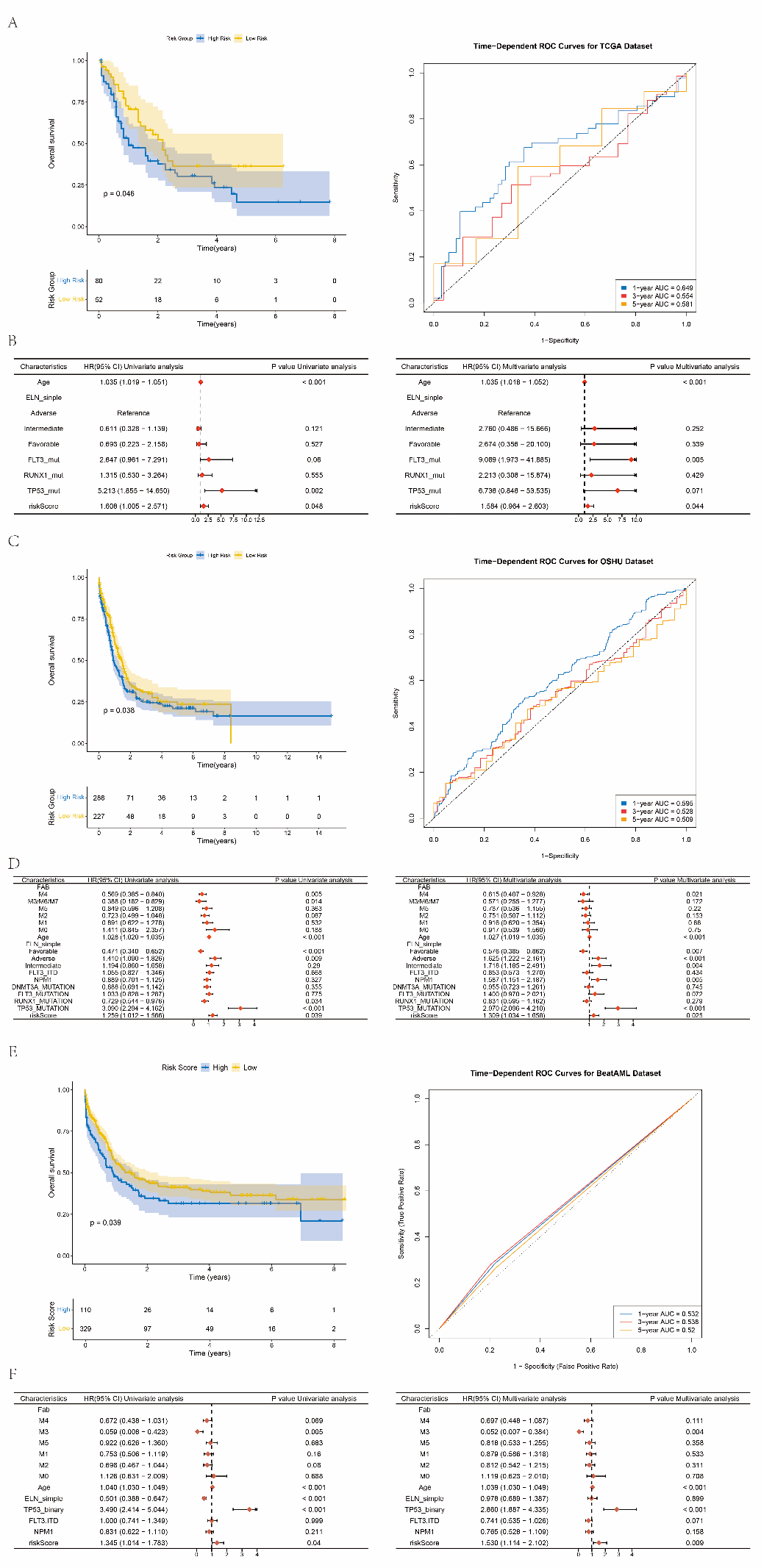
**

**Supplementary Figure S5. Validation of the** **RSF Model on Multiple Independent Cohorts**

(A) Kaplan-Meier survival analysis (high- and low-risk groups) and time-dependent ROC curves (1, 3, and 5 years) for the RSF model in the TCGA cohort.

(B) Forest plots of univariate and multivariate Cox regression analyses in the TCGA cohort for RSF model validation.

(C) Kaplan-Meier survival analysis (high- and low-risk groups) and time-dependent ROC curves (1, 3, and 5 years) for the RSF model in the OSHU cohort.

(D) Forest plots of univariate and multivariate Cox regression analyses in the OSHU cohort for RSF model validation.

(E) Kaplan-Meier survival analysis (high- and low-risk groups) and time-dependent ROC curves (1, 3, and 5 years) for the RSF model in the BeatAML cohort.

(F) Forest plots of univariate and multivariate Cox regression analyses in the BeatAML cohort for RSF model validation.

**
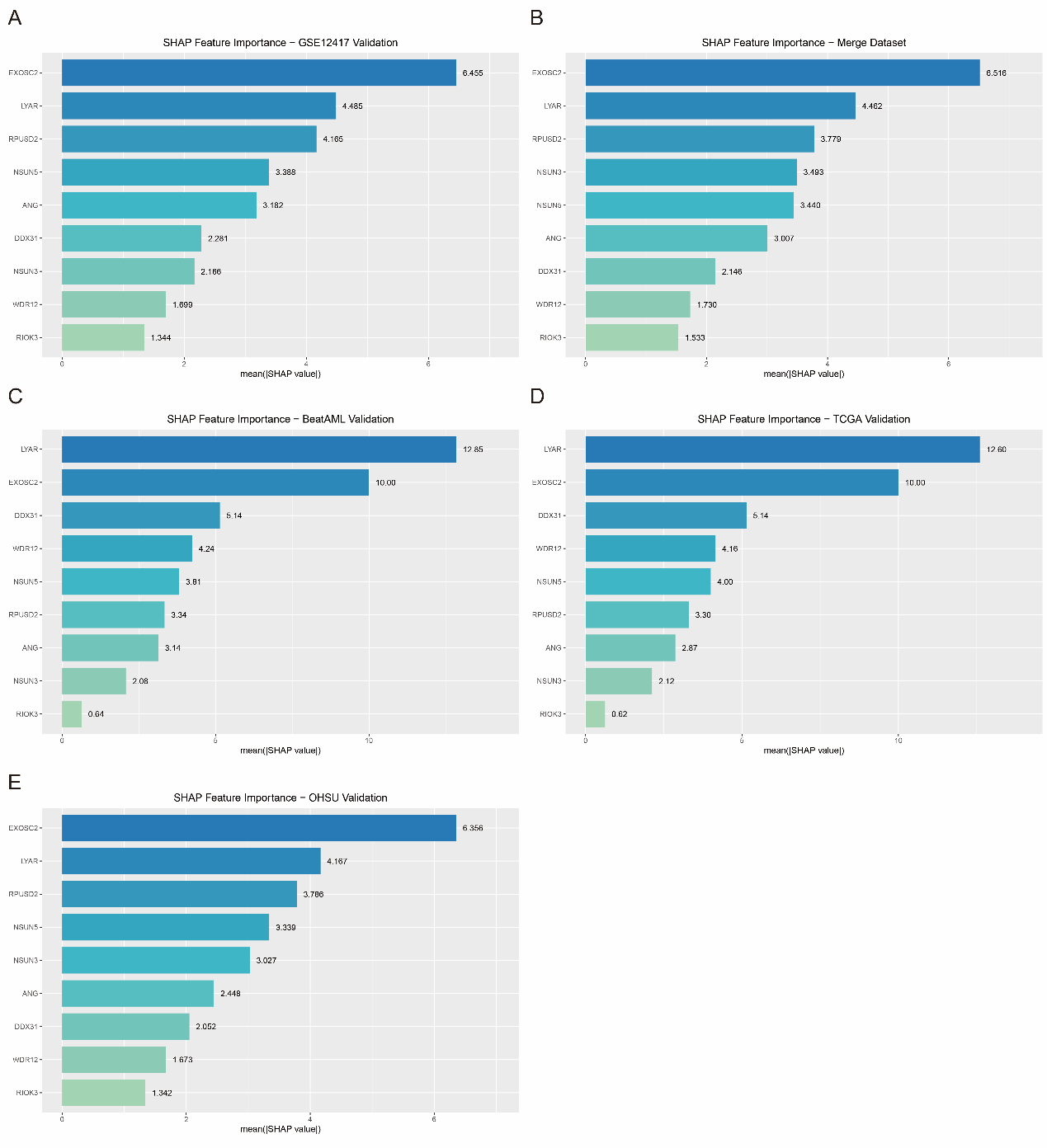
**

**Supplementary Figure S6. SHAP Feature Importance of 9 Genes Across Five Datasets**

A. SHAP Feature Importance – GSE12417 Validation

B. SHAP Feature Importance – Merge Dataset

C. SHAP Feature Importance – BeatAML Validation

D. SHAP Feature Importance – TCGA Validation

E. SHAP Feature Importance – OHSU Validation

**
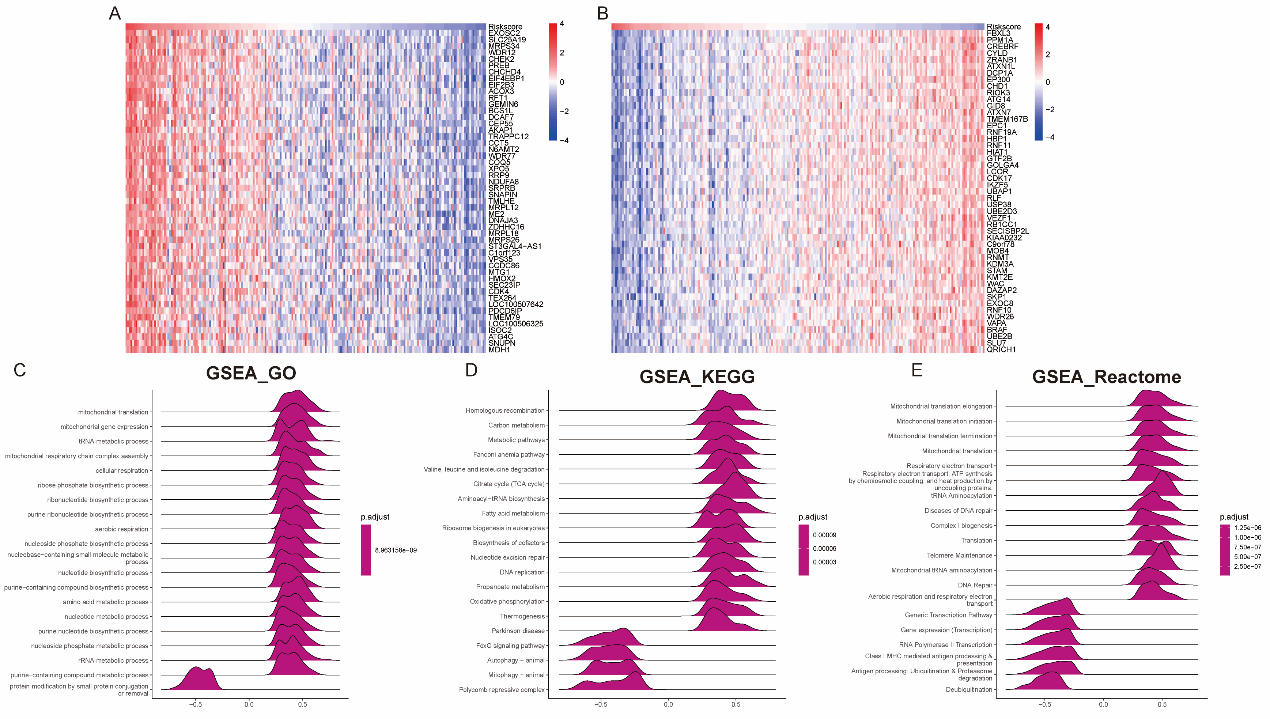
**

**Supplementary Figure S7. Screening and functional enrichment analysis of RBS risk score -associated genes**A-B. Correlation analysis between **RBS risk score** and all genes, visualized by heatmaps showing the expression patterns of the top 50 positively correlated genes (A), and the top 50 negatively correlated genes (B).

(C) GSEA_GO enrichment analysis.

(D) GSEA_KEGG enrichment analysis.

(E) GSEA_Reactome enrichment analysis.

**
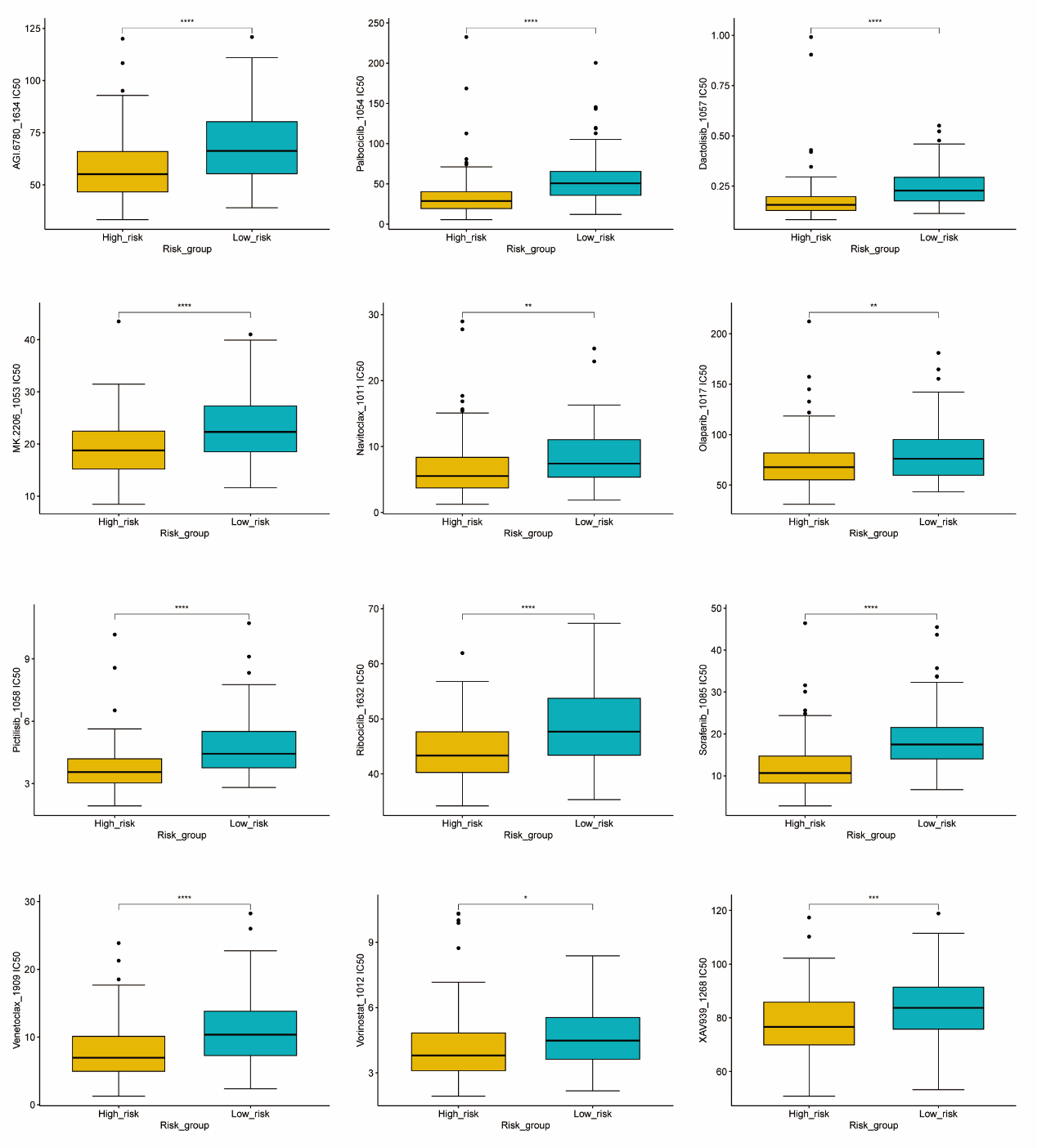
**

**Supplementary Figure S8. Drug Sensitivity Analysis**

Drug Sensitivity Analysis in Low-RBS and High-RBS Groups.
